# Genomic analysis of the multi-host pathogen *Erysipelothrix rhusiopathiae* reveals extensive recombination as well as the existence of three generalist clades with wide geographic distribution

**DOI:** 10.1186/s12864-016-2643-0

**Published:** 2016-06-14

**Authors:** Taya Forde, Roman Biek, Ruth Zadoks, Matthew L. Workentine, Jeroen De Buck, Susan Kutz, Tanja Opriessnig, Hannah Trewby, Frank van der Meer, Karin Orsel

**Affiliations:** Faculty of Veterinary Medicine, University of Calgary, Calgary, Alberta Canada; Current address: Institute of Biodiversity, Animal Health and Comparative Medicine, University of Glasgow, Glasgow, Scotland UK; The Roslin Institute, University of Edinburgh, Midlothian, Scotland UK

**Keywords:** *Erysipelothrix rhusiopathiae*, Genomics, Gram positive bacteria, Homologous recombination, Host specificity, Multi-host pathogen, Population structure, Whole genome sequencing

## Abstract

**Background:**

Knowledge about how bacterial populations are structured is an important prerequisite for studying their ecology and evolutionary history and facilitates inquiry into host specificity, pathogenicity, geographic dispersal and molecular epidemiology. *Erysipelothrix rhusiopathiae* is an opportunistic pathogen that is currently reemerging in both the swine and poultry industries globally. This bacterium sporadically causes mortalities in captive marine mammals, and has recently been implicated in large-scale wildlife die-offs. However, despite its economic relevance and broad geographic and host distribution, including zoonotic potential, the global diversity, recombination rates, and population structure of this bacterium remain poorly characterized. In this study, we conducted a broad-scale genomic comparison of *E. rhusiopathiae* based on a diverse collection of isolates in order to address these knowledge gaps.

**Results:**

Eighty-three *E. rhusiopathiae* isolates from a range of host species and geographic origins, isolated between 1958 and 2014, were sequenced and assembled using both reference-based mapping and *de novo* assembly. We found that a high proportion of the core genome (58 %) had undergone recombination. Therefore, we used three independent methods robust to the presence of recombination to define the population structure of this species: a phylogenetic tree based on a set of conserved protein sequences, *in silico* chromosome painting, and network analysis. All three methods were broadly concordant and supported the existence of three distinct clades within the species *E. rhusiopathiae*. Although we found some evidence of host and geographical clustering, each clade included isolates from diverse host species and from multiple continents.

**Conclusions:**

Using whole genome sequence data, we confirm recent suggestions that *E. rhusiopathiae* is a weakly clonal species that has been shaped extensively by homologous recombination. Despite frequent recombination, we can reliably identify three distinct clades that do not clearly segregate by host species or geographic origin. Our results provide an essential baseline for future molecular epidemiological, ecological and evolutionary studies of *E. rhusiopathiae* and facilitate comparisons to other recombinogenic, multi-host bacteria.

**Electronic supplementary material:**

The online version of this article (doi:10.1186/s12864-016-2643-0) contains supplementary material, which is available to authorized users.

## Background

Uncovering population structure and its determinants is essential for understanding bacterial ecology and evolution [1]. Key questions concerning host specificity and generalism [2], global patterns of gene flow [3], and the genetic basis for clinical disease manifestations [1] can be addressed by examining the relationships among strains within a species. Additionally, molecular epidemiological studies rely on having an understanding of the population structure as a framework within which to interpret the genomic diversity of the target organism [4], and the identification of host- or geography-associated lineages can be helpful in source attribution [5]. Whole genome sequencing is providing new opportunities to address questions related to population structure within a phylogenetic framework; however, this undertaking is complicated by the need to consider the potential influence of recombination [4, 6].

*Erysipelothrix rhusiopathiae*, a Gram positive, facultative intracellular bacterium, is an important opportunistic pathogen for both humans and animals. Zoonotic infections with *E. rhusiopathiae* typically manifest as erythematous skin lesions known as erysipeloid, and tend to be occupationally associated (e.g. slaughterhouse workers, butchers, fishermen, etc.) [7]. *E. rhusiopathiae* has been documented in a wide range of wild and domestic species, including birds, mammals, reptiles, fish and arthropods [8]. Best known as the causative agent of swine erysipelas, *E. rhusiopathiae* can cause significant economic losses in swine production systems due to sporadic cases of acute septicemia, subacute cutaneous lesions, or chronic arthritis, which may be punctuated by larger outbreaks [9]; it is also among the most common causes of carcass condemnation for swine in the United States [10]. In recent years, the incidence of *E. rhusiopathiae* infection in swine has increased significantly in the mid-western United States, Japan and China [10–12]. Erysipelas is also reemerging in European poultry productions, likely in association with changes in housing systems [13, 14]. In captive marine mammals, *E. rhusiopathiae* is known to cause serious and often life-threatening infections [15, 16], while recent die-offs involving hundreds of muskoxen in the Canadian Arctic Archipelago have sparked interest in the potential conservation importance of this bacterium [17]. The broad ecological and geographic distribution of *E. rhusiopathiae* has been attributed to its ability to infect multiple host species which may act as healthy carriers, in combination with its long environmental persistence [18]. However, despite its ubiquity, importance for multiple host species including humans, and a highly variable clinical presentation, little is known about the genetic diversity, population structure, and host specificity of *E. rhusiopathiae*.

Recent whole genome sequencing projects have facilitated the taxonomic classification of the genus *Erysipelothrix* [11, 19]*.* A member of the phylum Firmicutes, the class Erysipelotrichia has the single order Erysipelotrichales and family Erysipelotrichaceae, the latter comprising 10 genera [20]. Within the genus *Erysipelothrix*, other recognized species are *E. tonsillarum, E. inopinata*, *E.* sp. strain 1, and *E.* sp. strain 2 [21, 22], as well as the recently identified *E. larvae* sp. nov. [23]. *E. rhusiopathiae* and *E. tonsillarum* have long been recognized as distinct species based on differences in pathogenicity, phenotypic characteristics, and serotype groups [21, 24], supported by DNA-DNA hybridization studies [25]. *E. inopinata* appears to have diverged prior to the split between *E. rhusiopathiae* and *E. tonsillarum* based on 16S rRNA gene sequence phylogeny [22]. The relationship of *E.* sp. strain 1 and *E.* sp. strain 2 to other *Erysipelothrix* species has not been explored.

The intraspecific classification of *E. rhusiopathiae* strains is less clearly defined, with a variety of different tools used for categorizing isolates. Serotyping, which involves testing for agglutination with specific antisera recognizing different peptidoglycan antigens of the cell wall [26], is one of the approaches that has been most frequently implemented. Although it has diagnostic value, as the different *Erysipelothrix* species have distinct sets of serotypes [27], serotyping is an inappropriate tool for inferring evolutionary relatedness among isolates due to the frequent horizontal exchange of capsule-specific genes in many bacterial species [28]. More recently, differences in immunogenic proteins known as surface protective antigens (Spa) have also been used to distinguish between strains of *E. rhusiopathiae* [13, 29]. The genetic relationship among *E. rhusiopathiae* isolates has also been examined using various comparative genotyping methods such as pulsed-field gel electrophoresis (PFGE) [30]. However, sequence-based typing methods, which allow for easier inter-laboratory comparison and investigation of functional differences, have only very recently been applied to *E. rhusiopathiae*: a comparison was made among the three *E. rhusiopathiae* whole genome sequences (two complete and one draft) available on GenBank at the time of writing [11], and the population structure of this bacterium was examined using multi-locus sequence typing (MLST), focusing primarily on European poultry isolates [13]. Little is known about the importance of recombination in *E. rhusiopathiae*, although the latter study found the species to be weakly clonal [13], suggesting recombination may contribute significantly to genetic variability in this species. Until now there have been no large-scale genomic comparison studies to fully investigate the genetic diversity of *E. rhusiopathiae*.

The objectives of this study were to describe the global genomic diversity of *E. rhusiopathiae* and to examine its population structure while accounting for the presence of recombination, in order to provide an essential baseline for future studies into the epidemiology and ecology of this multi-host pathogen. A secondary objective was to examine the phylogenetic relationship among different *Erysipelothrix* species based on whole genome sequence data.

## Results

### Sequencing and pan-genome statistics

Eighty-three newly sequenced *E. rhusiopathiae* isolates were included in this analysis, with representation from North America (Canada and US), South America (Argentina), Europe (Belgium, Hungary and the UK), Asia (Japan), South Africa, and Australia, with the majority originating from Canada (*n* = 37), US (*n* = 20) and Belgium (*n* = 17) (see Additional file 1: Table S1 for a table of the isolates included in this study). Dates of isolation ranged from 1958 to 2014. Host species of origin encompassed swine (*n* = 18), poultry (*n* = 14), captive marine mammals (dolphins and beluga whales; *n* = 8), fishes (*n* = 5), wild birds (primarily water fowl; *n* = 7), wild ungulates (muskoxen, moose and caribou; *n* = 28), and a wolf. Background information regarding the clinical manifestations associated with the isolates was only available for 12 isolates (Additional file 1: Table S1), but cases of acute and subacute septicemia, as well as *E. rhusiopathiae* isolated from skin lesions were included. Serotype was known for 16 isolates previously described in the literature (Additional file 1: Table S1); the newly serotyped isolate VI11-2_lu was found to belong to serotype 5.

**Fig. 1:**
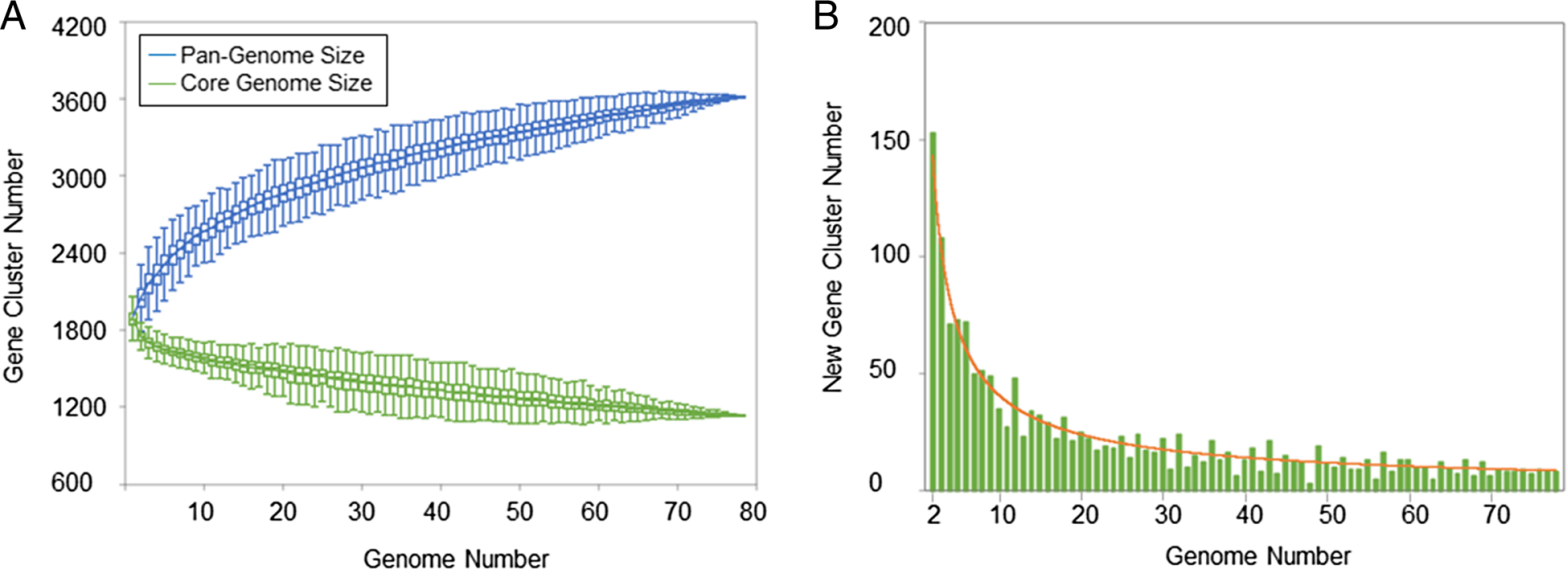
*Erysipelothrix rhusiopathiae* pan-genome. Plots were generated in LS-BSR and visualized using PanGP. **a** Convergence of the core genome with concurrent accumulation of coding sequences in the pan-genome in relation to the number of genomes analyzed. **b** Number of unique coding sequences for each additional genome analyzed. Publicly available *E. rhusiopathiae* isolates and *de novo* assembled isolates whose average depth of coverage was greater than 15X were included in this analysis

A total of 1137 core genes were present among all the *E. rhusiopathiae* isolates included in this analysis, representing 67 % of the coding sequences present in the Fujisawa reference genome (Fig. 1a and Additional file 2: Figure S1). A total of 512 singleton genes were identified (i.e. present in only one isolate), with an average of 6.6 new unique genes discovered per additional genome sequenced (Fig. 1b). Among the core genes were the *ComEC* (membrane pore) and *dprA* (recombination mediator) genes, as well as putative Type II/IV secretion system proteins (Additional file 2: Figure S1), the presence of which is highly suggestive of natural competency [31]. Based on the pairwise comparisons made in the program LS-BSR, no major differences in overall gene content were detected among host species or geographic origin, or among clades that were identified during analyses described below.

### A high proportion of the *E. rhusiopathiae* core genome has experienced recombination

The core genome alignment generated using Parsnp was 1,049,431 base pairs, representing 58 % of the reference genome. Overall there was a high degree of sequence similarity, with 99.3 % pairwise identity. The BratNextGen analysis, which included all isolates, inferred that 58 % of the overall core genome alignment had experienced recombination (Additional file 2: Figure S2). There were major differences among the clades in terms of the proportion of the alignment with inferred recombination (Table 1, Additional file 2: Figures S2 and S3). Both recombination detection methods estimated that a greater proportion of the genome of Clade 2 isolates has experienced recombination. BratNextGen did not detect any recombinogenic segments in Clade 1, while this was also the clade with the least recombination across the core genome as detected by Gubbins. Although a large proportion of the alignment was influenced by recombination, average recombination to mutation (r/m) rates were moderate, at 0.96, 2.18 and 0.55 in Clades 1, 2 and 3 respectively.

**Table 1:** Recombination detected in the core genome of *Erysipelothrix rhusiopathiae*

	Clade 1	Clade 2	Clade 3
% of alignment implicated BNG	0	38	24
% of alignment implicated Gubbins	27	86	58
r/m	0.96	2.18	0.55

Percentage of the *Erysipelothrix rhusiopathiae* core genome found to have experienced recombination within each clade using BratNextGen (BNG) and Gubbins. Recombination to mutation (r/m) rates for each clade were estimated in Gubbins

### Phage and plasmid-related sequences were detected in several isolates

Putative prophage sequences were detected in 47 of the 86 isolates (55 %), representing all host groups and geographic locations (Additional file 1: Table S1). Up to three such sequences were detected per isolate, with a mean length of 22 KB (range: 12.5–47 KB). Twenty-eight of the isolates had phage sequences with a high level of homology (≥96.5 % pairwise identity) with the annotated bacteriophage from the Fujisawa genome (36.5 KB; Additional file 2: Figure S1) across at least an 8 KB segment (designated as P1 in Additional file 1: Table S1). Two of these 28 isolates were inferred to be intact prophages by PHAST. Isolates with P1 phage sequences were present in all three clades, suggesting that the presence of these similar phage sequences is the result of either multiple introductions or ancestral acquisition with subsequent loss along various branches of the phylogeny. A second group of phage sequences (*n* = 15; designated as P2 in Additional file 1: Table S1) shared 95.9 % pairwise identity across about 12 KB. Four of these were classified as intact by PHAST. There is a strong possibility that some of these P2 sequences have been inherited through vertical descent (e.g. in the five Belgian swine isolates), while the other sequences were identified in unrelated isolates, indicating horizontal transfer. Eight isolates had phage sequences that clustered into a third group (P3) that had at least 12 KB of homologous sequence with 94 % pairwise identity. None of these sequences were designated as intact by PHAST. These sequences all belonged to swine isolates from two distinct clusters, although not all isolates in each cluster had phage sequences detected. Among the other phage sequences detected, most shared a smaller portion of sequence homology with one of the three groups of sequences (designated as ‘partial’ in Additional file 1: Table S1). Only one unique phage sequence was identified among our collection using PHAST: the 22 KB incomplete phage sequence in the poultry isolate G11. Based on BLAST comparisons, this sequence shares a 13 KB segment with an integrative conjugative element of *Streptococcus pyogenes* with 95.4 % pairwise identity, while the rest of the sequence shared 96 % pairwise similarity to plasmids carrying coding sequences for Type IV secretory pathway components.

The BLAST search against the publicly available *E. rhusiopathiae* plasmid sequence [GenBank: NC_002148] found that one isolate in our collection (‘Ery Afrika 1’, Clade 2) had a similar sequence, with 95.2 % pairwise identity and 100 % coverage. During the BLAST search of contigs from each isolate that did not align to the reference genome, one poultry isolate had a hit to a conjugal transfer protein, while five swine isolates had hits to plasmid sequences. Three of these, from swine from Belgium, were hits to the same plasmid sequence; searches for similar sequences among the other three Belgian swine isolates were negative.

### Population structure of *E. rhusiopathiae*

**Fig. 2:**
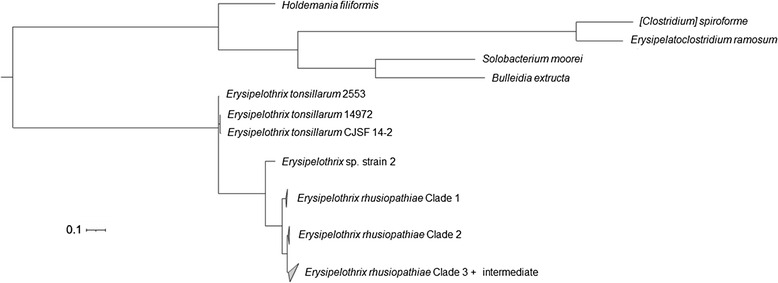
Relationship among the species of the genus *Erysipelothrix*. This phylogenetic tree is based on > 400 conserved bacterial protein sequences, generated using PhyloPhlAn. Members of other genera of the family Erysipelotrichaceae were used as the outgroup for rooting the tree. The scale bar represents the expected number of nucleotide substitutions per sequence position

**Fig. 3:**
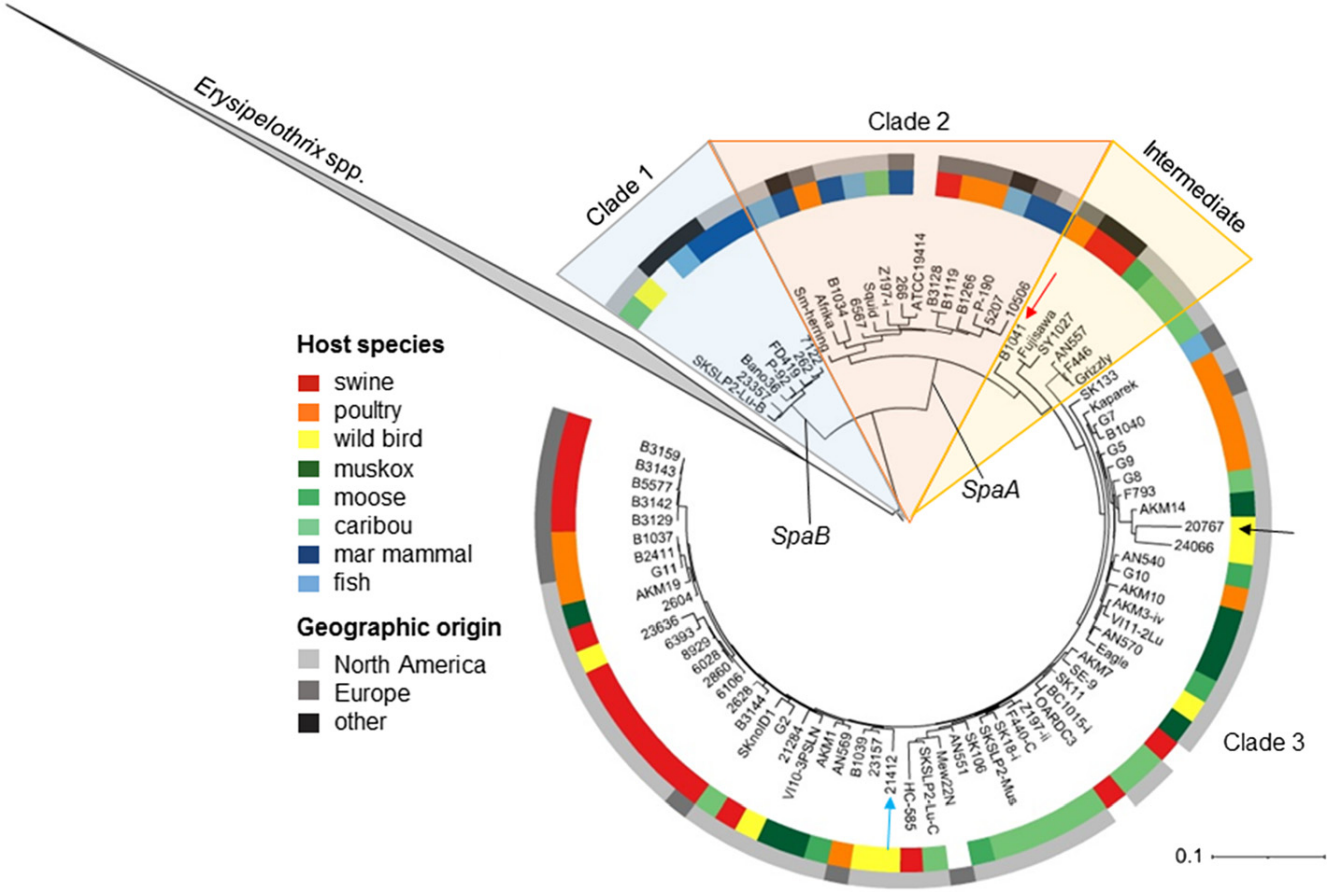
Population structure of *Erysipelothrix rhusiopathiae* based on phylogenetic inference. This tree is based on > 400 conserved bacterial protein sequences, generated using PhyloPhlAn. Other *Erysipelothrix spp.* were used as the outgroup for rooting the tree (*clade collapsed*). Concentric rings illustrate host species of origin (*colored squares*) and geographic origin (*grey-scale*). Arrows indicate isolates whose correct clade association is not resolved between the phylogenetic and chromosome painting approaches. Presence of the surface protective antigen type B (*SpaB*) gene was exclusive to Clade 1, while the *SpaA* gene was found among the two other clades and intermediate isolates

**Fig. 4:**
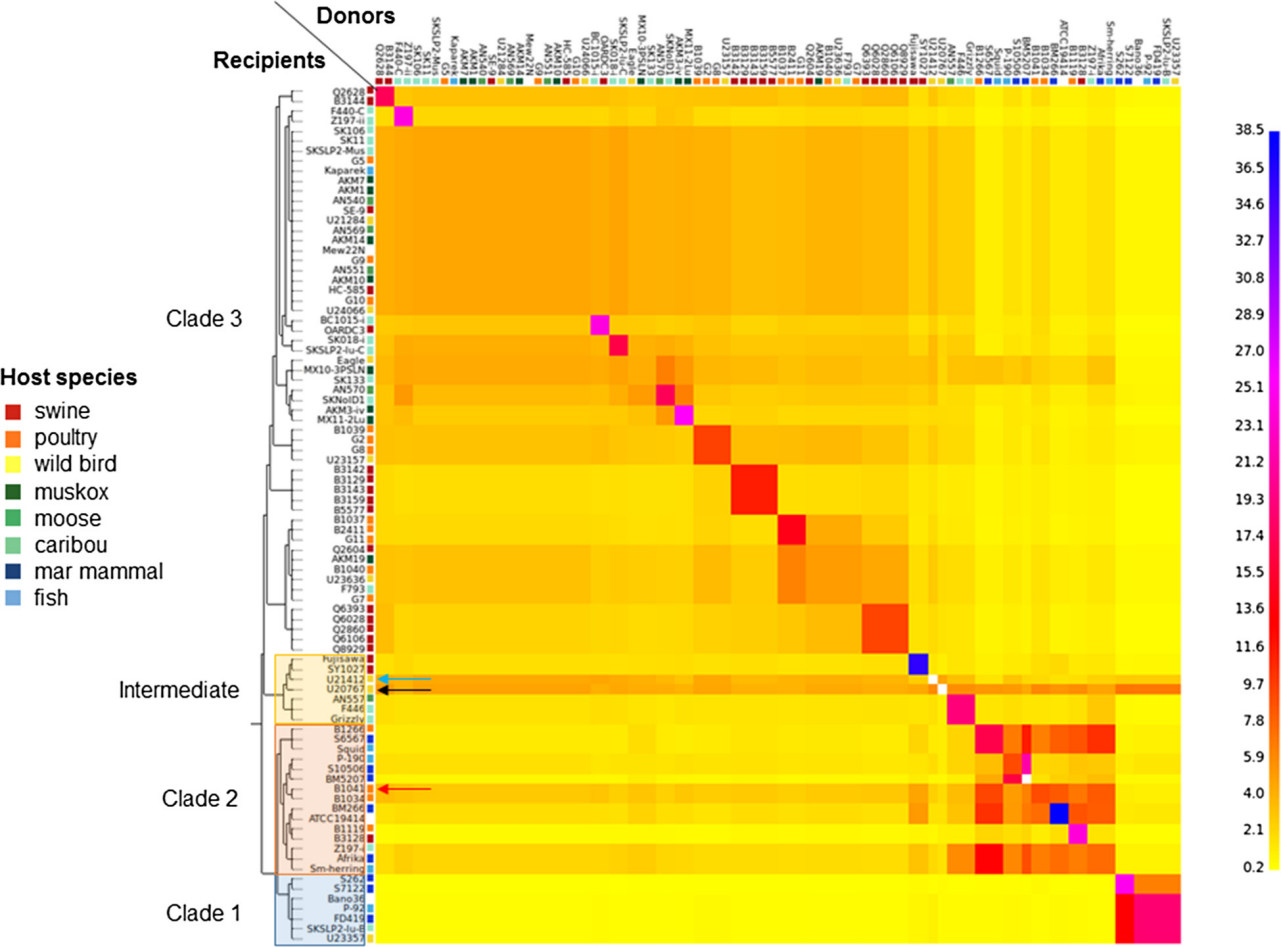
Population structure of *Erysipelothrix rhusiopathiae* based on *in silico* chromosome painting. Tree and heat map illustrate the relatedness among *E. rhusiopathiae* isolates based on chromosome painting using ChromoPainter and model-based clustering using Bayesian Markov chain Monte Carlo (MCMC) analysis in fineSTRUCTURE. The color scale represents the number of ‘chunks’ shared between populations of donors (*x-axis*) and recipients (*y-axis*). Population subgroup assignment is shown in Figure S4 in Additional file 2. Arrows indicate isolates whose correct clade association is not resolved between the phylogenic and chromosome painting approaches

**Fig. 5:**
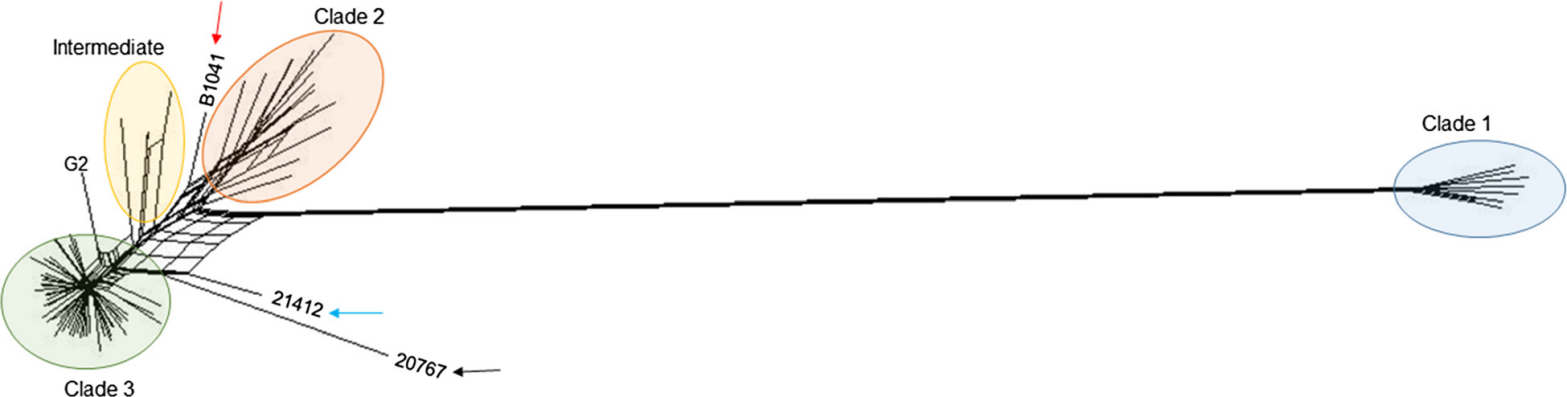
Population structure of *Erysipelothrix rhusiopathiae* based on network analysis. A phylogenetic network of *E. rhusiopathiae* was estimated using Neighbor-Net analysis as implemented in SplitsTree. Arrows indicate isolates whose correct clade association is not resolved between the phylogenic and chromosome painting approaches

The phylogenetic tree generated in PhyloPhlAn using > 400 conserved bacterial proteins, rooted to other genera of the family Erysipelotrichaceae*,* places *E. tonsillarum* at a position basal to *E. rhusiopathiae* and *E.* sp. strain 2 in the phylogeny (Fig. 2). The differentiation between clades is more easily visible when zooming in to the within-species level (Fig. 3). Consistent with this phylogeny, the analysis using ChromoPainter and fineSTRUCTURE (Fig. 4 and Additional file 2: Figure S4), and network analysis implemented in SplitsTree (Fig. 5) supported the existence of three distinct clades within the species *E. rhusiopathiae*. Seven isolates belonged to Clade 1 as supported by all three methods. These isolates originated from two captive marine mammals (beluga and dolphin) from the US, one dolphin from an Australian aquarium, one red wolf from the US, one fish from Japan, one caribou from Canada, and from a sheep dip from Argentina. Fourteen isolates belonged to Clade 2 as supported by all three methods. These consisted of isolates from marine mammals (*n* = 5) from the US, Belgium and South Africa, from fish (*n* = 3) from Japan and the US, from poultry (*n* = 3) and swine (*n* = 1) from Belgium, from one Canadian caribou and the ATCC19414 isolate from GenBank (host species and geographic origin unknown; see Additional file 1: Table S1). One additional poultry isolate from Belgium (red arrow Figs. 3, 4 and 5) was grouped with the Clade 2 isolates by ChromoPainter, but was classified as an “intermediate” isolate between Clades 2 and 3 using phylogenetic analysis. At least five isolates clustered in this intermediate group based on all three methods: the two whole genome sequences from swine available on GenBank (SY1027 from China and Fujisawa from Japan), and two caribou and one moose isolate from Canada. By the chromosome painting method, two additional isolates clustered within this intermediate clade, both from wild birds from the US; these isolates fell within Clade 3 using the phylogenetic approach (blue and black arrows in Figs. 3, 4 and 5). The three isolates whose clade designation differed between the phylogenetic and chromosome painting methods all fell outside of the main clusters using network analysis (Fig. 5, shown by arrows). The other 57 isolates constituted the dominant Clade 3, composed of all of the Canadian swine (*n* = 8) and poultry (*n* = 6) isolates tested, most of the Belgian swine isolates (6/7) and half of the Belgian poultry (4/8) isolates, three additional swine isolates (one from the US and two of unknown origin), isolates from five different North American wild bird species, isolates from North American caribou (*n* = 11), moose (*n* = 4) and muskoxen (*n* = 8), and one fish isolate from Hungary.

**Fig. 6:**
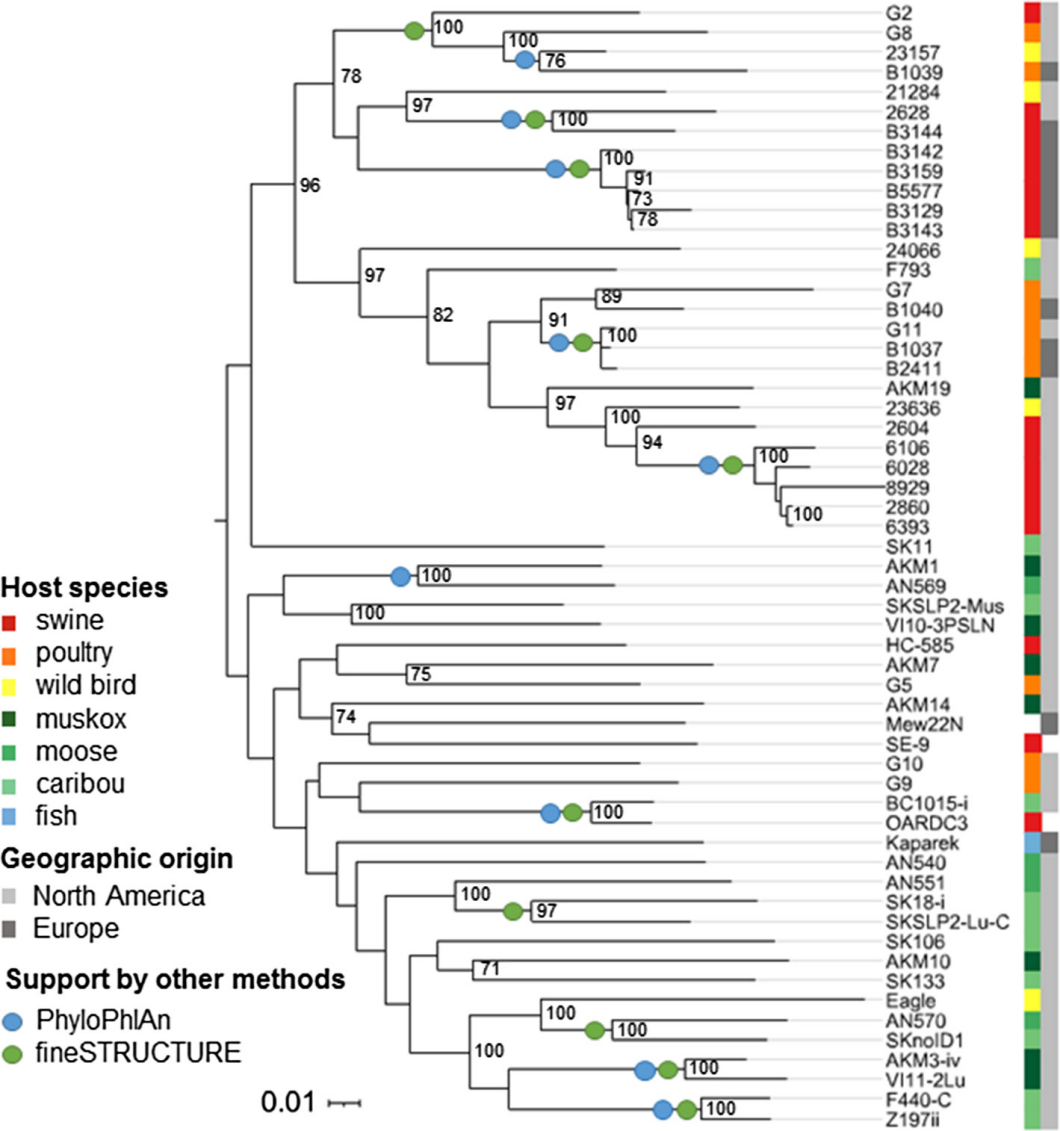
Relationship among Clade 3 isolates. This maximum likelihood tree (mid-point rooted) was generated using PhyML based on the curated set of single nucleotide polymorphisms (SNPs) found to be outside recombinant segments as determined using Gubbins. Bootstrap values with >70 % support are shown, in addition to support for clusters of isolates by PhyloPhlAn and fineSTRUCTURE

Less concordance was observed among the different methods with respect to the relationships of isolates within the dominant Clade 3. This is illustrated in Fig. 6, which shows the maximum likelihood (ML) phylogeny generated using non-recombinogenic core single nucleotide polymorphisms (SNPs) inferred by Gubbins, with clusters supported by either PhyloPhlAn or fineSTRUCTURE superimposed. In general there was stronger support for more recent nodes based on higher bootstrap values in the ML tree, as well as more frequent support by the other two methods. No temporal signal was detected in the root-to-tip analysis of Clade 3 isolates using Path-O-Gen (correlation < 0.001).

A consistent relationship was found between Spa*-*type and clade: all isolates in Clade 1 had a single copy of the *SpaB* gene, while all other *E. rhusiopathiae* isolates had a single copy of *SpaA* (Fig. 3). Conversely, among those isolates whose serotype had been defined, significant homoplasy was observed (Additional file 2: Figure S5). Serotype 5 isolates were found in all three clades, serotype 2 isolates were found in Clades 2 and 3, and serotype 1a isolates fell into both intermediate and Clade 3.

### Limited evidence for host or geographic specificity

All three clades included isolates from various host species and from multiple continents. However, all marine mammal isolates (*n* = 8) fell into Clades 1 or 2 and none within the dominant Clade 3, while no swine or poultry isolates were found to belong to Clade 1. Within Clade 3, clustering by host species, geographic location, and submitting laboratory were all found to be significant using the program BaTS (Additional file 3: Table S2). Although not fully supported by the other two methods, the phylogenetic tree based on non-recombinant SNPs (Fig. 6) grouped the majority of livestock isolates within Clade 3 (21/27 swine and poultry isolates) into a single sub-group with 96 % bootstrap support, whereas the majority of isolates of wildlife origin fell into a separate sub-group within this clade.

## Discussion

### Homologous recombination has occurred extensively throughout the core genome of *E. rhusiopathiae*

This is the first large-scale genomic study of the multi-host pathogen *E. rhusiopathiae*. Our results indicate that homologous recombination plays an important role in generating diversity within this species, confirming recent findings by an MLST study [13]. Based on our data, several mechanisms may contribute to recombination in this species: the uptake of genetic material from the external environment, phages, and plasmids. Bacteria capable of importing foreign DNA across the cell envelope (transformation) are said to be ‘naturally competent’ and possess specific machinery for this task [31]. The presence of the *ComEC* (membrane pore) and *dprA* (recombination mediator) genes within the core genome, as well as coding sequences for putative Type II/IV secretion system proteins, is highly suggestive that *E. rhusiopathiae* has this capacity [31, 32]. Transduction and conjugation may also be important mechanisms for horizontal gene transfer in *E. rhusiopathiae*, since phage-related sequences were abundant among the isolates in our study and some plasmid-related sequences were also identified, despite the fact that the sensitivity for detecting such sequences from draft assemblies may be low [33]. In previous studies, up to a third of isolates tested were found to harbor plasmids [34, 35], and several mobile genetic elements in the available reference genomes bore signatures of having plasmid or prophage origin [11]. To our knowledge, this is the first study to have examined the prevalence and diversity of phage-related sequences in a large collection of *E. rhusiopathiae* isolates. Although limited data were available regarding the clinical presentation associated with many of the isolates, no clear association was found between the presence of phage sequences and pathogenicity. The presence of particular phage sequences did not appear to be a phylogenetically informative marker for epidemiological inference, since related phage sequences were found throughout the different clades, and in some cases, phage sequences were not conserved within groups of highly similar isolates. Similarly, the inconsistent presence of plasmid sequences among closely related isolates suggests that conjugation may occur frequently.

Although a high proportion of the core genome has experienced recombination events, the r/m rates we estimated for each Clade (between 0.55 and 2.18) were only moderate in comparison to other bacterial species [36]. This, along with the high pairwise identity (99.3 %) found within the core genome, suggests that within the *E. rhusiopathiae* core genome, alleles are highly conserved, although its organization is not. This may explain the lack of concordance found between MLST and PFGE [13], since this latter technique is sensitive to structural rearrangements. Within each clade, a larger proportion of the core genome was estimated to have experienced recombination based on the Gubbins output in comparison to BratNextGen. Gubbins is based on the method initially cited in Croucher et al. [37], and has since been employed in several other studies of bacterial pathogens [38, 39]. Inference of recombinant segments is based on the detection of areas of higher SNP density in comparison to the background threshold level. Since areas of elevated mutations are not necessarily specific for recombination and could instead represent regions of the genome with naturally higher mutation rates or that are under less purifying selection, this could be a potential source of false positives using this method [40]. BratNextGen, on the other hand, uses a Bayesian change-point clustering model to detect evolutionarily distinct lineages; these clusters are taken as the putative ancestral origins when estimating recombination probabilities in each isolate. This program has been frequently cited in intraspecific bacterial population studies based on whole genome data [41, 42]. Despite the differences in the extent of recombination found between the two programs used, both suggest that recombination has occurred most frequently in Clade 2, where the r/m rate was two to four times higher compared to the other clades and a greater proportion of the genome was found to have been implicated. Clade-associated differences in recombination rates have been previously observed in other bacterial species such as *Listeria monocytogenes* [43].

### The population structure of *E. rhusiopathiae* was consistent across multiple inference approaches

Given the uncertainties associated with whole genome phylogenetic reconstruction in the presence of recombination, we took three conservative, independent approaches for inferring the population structure of *E. rhusiopathiae.* The use of a set of conserved protein sequences has been shown to be robust to horizontal gene transfer [44]. Chromosome painting does not rely on phylogenetic inference but rather reconstructs the chromosome haplotype of “recipient” individuals as a composition of recombination-derived segments from the other “donor” individuals [45]. Finally, network analysis provides a means by which to visualize alternative phylogenetic histories in organisms that are not strictly clonal [46]. Using these three approaches, good concordance was found for assigning isolates into clades. The fact that these methods were based on input from both *de novo* genome assembly (conserved protein tree and network analysis) and a reference-based mapping approach (chromosome painting), provides support for the robustness of these results.

Clade 1 could be distinguished from the other two clades and intermediate isolates by the presence of a *SpaB* gene, as opposed to *SpaA* found in the other isolates. These surface protective antigens are potent immunogens with potential relevance for vaccine development [29]. *SpaA* is among the more well-characterized genes that have been proposed to be associated with *E. rhusiopathiae* pathogenicity [19], with a role in endothelial adherence [47] and resistance to phagocytosis [48]. The *SpaB* gene shares approximately 60 % amino acid similarity with *SpaA* and is antigenically distinct [49]. It was previously found that *E. rhusiopathiae* strains of aquatic origin were able to express more than one Spa*-*type [29], although an underlying genetic basis for this was not observed among the isolates in our collection. Not surprisingly, serotype was not associated with the phylogenetic relatedness among isolates [50, 51]. Since homoplasy of serotype is likely commonplace (Additional file 2: Figure S5), it should not be used to infer evolutionary relatedness among isolates, and its use as an epidemiological tool (e.g. for deciding whether multiple isolates belong to the same outbreak) would require additional information on the expected frequency of serotype switching.

Despite removing recombinogenic sites prior to constructing the phylogeny of the dominant Clade 3, basal relationships among these isolates were difficult to elucidate, with low bootstrap values obtained for most of the deeper nodes (Fig. 6). It is possible that not all recombination events were detected and that this still had a confounding influence; a PHI-test revealed a strong signal of recombination even in this curated SNP set. The lack of a detectable clock-like signal could be partially due to residual recombinogenic segments in this dataset [4], although given the lack of correlation between the date of isolation and root-to-tip divergence, it seems probable that additional factors are contributing. Hypothetically, highly variable generation times (e.g. little bacterial replication during environmental phases or carriage in comparison to during active infection) could be a plausible explanation for the lack of temporal structure. Failing to calibrate the molecular clock, we were unable to estimate a global or clade-specific substitution rate for *E. rhusiopathiae* based on whole genome sequence data.

### *E. rhusiopathiae* shows limited host association

Bacteria show remarkable variability in the extent to which they specialize to colonize specific host species, the determinants of which are thought to be the result of complex host-pathogen interactions [52]. Many bacterial species have fairly distinct host-adapted strains [2, 53], while other bacterial species are characterized by a mix of host-adapted and generalist sub-types [42, 54]. The determinants of host specificity can be seen along a broad continuum from changes in a single amino acid residue, to changes in gene content (presence/absence) or the acquisition of genomic islands [55]. In our study, no consistent differences in gene content were found between isolates from different host species, geographic locations or clades.

It is known from experimental infection studies that multiple host species are susceptible to the same strains of *E. rhusiopathiae*; isolates from one host species that are subsequently inoculated into another species often result in infection and clinical disease in the recipient species [56], although susceptibility varies [57]. We found that all three clades comprised isolates from a variety of taxonomically very different host species. It is, therefore, probable that all *E. rhusiopathiae* lineages are infectious for a wide range of species, although possibly with varying levels of infectiousness and pathogenicity. Our data also contained strong indications of cross-species transmission given that some isolates from wildlife were nested within lineages dominated by domestic hosts and vice versa (Figs. 3 and 6), suggesting host or ecological predilection exists, but not strict host specificity. Similar findings were reported using MLST, wherein the dominant clonal complex encompassed isolates from multiple host species, including poultry, pigs, sheep and humans [13].

Although the different clades do not appear to be strictly limited to specific hosts, the observation that isolates from captive marine mammals fell exclusively into Clades 1 and 2 is significant (*p* < 0.0001), especially given that these are from aquariums distributed globally. Whether this finding is associated with differences in susceptibility of marine mammals to these strains, or that these strains are differentially distributed due to adaptions to marine environments warrants further investigation. Both Clade 1 and Clade 2 *E. rhusiopathiae* isolates were associated with fatal infections in marine mammals (Additional file 1: Table S1). However, no Clade 1 isolates, and therefore no strains carrying the *SpaB* gene, were found in any of the swine or poultry isolates in our collection. Since it has been previously shown that strains of marine origin carrying the *SpaB* gene can experimentally cause at least mild to moderate lesions in swine [56], the lack of Clade 1 strains in swine and poultry is more likely due to lack of exposure than a lack of pathogenic potential.

Within the dominant Clade 3, although clustering by host species was detected, equally strong support for clustering based on geographic origin and source laboratory was found (Additional file 3: Table S2), suggesting that these associations cannot be separated from sampling bias related to the opportunistic nature of our isolate collection. This represents a common challenge in phylogenetic studies of pathogens [58]. Further examination into host and geographic clustering within this clade using a broader set of sample sources, including more detailed metadata in order to rule out clustering due to epidemiologically linked isolates, would be of value. Despite this limitation, there appears to be a non-random segregation of Clade 3 isolates between those of livestock and wildlife origin (Fig. 6); swine and poultry isolates from three independent sources of both North American and European origin tended to cluster independently of those from northern ungulates. Among the livestock isolates, there was evidence of regular cross-continental exchange.

Although efforts were made to include a global collection of isolates from a representative variety of host species, most isolates originated from North America and Europe, and some important hosts including humans, reptiles and arthropods were not part of our collection. The characterization of additional isolates, particularly from under-represented continents and host species, will help build our understanding of the global population structure of *E. rhusiopathiae.*

### Whole genome sequencing supports the current division among *Erysipelothrix* species

We have shown *E.* sp. strain 2 to be phylogenetically distinct from both *E. rhusiopathiae* and *E. tonsillarum* based on the conserved protein phylogeny. Its distinction from *E. rhusiopathiae* is further supported by the fact that its level of sequence similarity was insufficient for mapping of reads to the full genome sequences of either Fujisawa or SY1027, or for inclusion in the core genome alignment generated through Parsnp, which requires ≥ 97 % average nucleotide identity. Based on the conserved protein phylogeny, *E. rhusiopathiae* and *E.* sp. strain 2 appear to have evolved from the less pathogenic species *E. tonsillarum*. This relationship was not observed in previous phylogenetic studies that combined the output from multiple phylogenetic approaches [20]. Further investigation into the genetic differences separating *E. rhusiopathiae* and *E.* sp. strain 2 from *E. tonsillarum* could potentially provide insights into how these species have acquired a higher level of pathogenicity. The recent detection of *Erysipelothrix* spp. in the subsurface biosphere may represent an interesting opportunity for examining the evolution of this genus in a broader context [59].

## Conclusions

Using different approaches, we were able to confidently determine the population structure of the multi-host pathogen *E. rhusiopathiae*, despite the important role that recombination has had in its evolutionary history. Evidence was found to suggest that novel DNA may be acquired by this bacterium through transformation, transduction and conjugation, lending plasticity to the genome. The species comprises three major clades that are found across multiple continents and host species representing both livestock and wildlife, with some indication that clades or subclades may differ in their host predilection and recombination rate. Epidemiological inference was hampered by the opportunistic nature of the isolate collection available for genome sequencing and future studies would benefit from targeted sample collection. Nonetheless, our results provide an essential framework for supporting future in-depth epidemiological and evolutionary studies involving this species and comparative studies with other recombinogenic, multi-host bacteria.

## Methods

### Bacterial isolates

In order to examine the intraspecific genomic diversity of *E. rhusiopathiae*, isolates were opportunistically collected so as to encompass a broad range of geographic locations, host species, clinical manifestations, and years of initial isolation. *E. rhusiopathiae* isolates, as well as isolates from other *Erysipelothrix* spp. (*E. tonsillarum* and *E.* sp. strain 2) were kindly provided by various collaborators (Additional file 1: Table S1). Upon receipt, isolates were sub-cultured onto Columbia Agar (CA) with 5 % sheep blood (BD-Canada, Mississauga, ON, Canada) for morphological characterization; if colony morphology was characteristic of *E. rhusiopathiae* (clear to pale blue in color, circular, small diameter, often with alpha hemolysis [18]), a single colony was re-streaked to obtain a clonal population for DNA extraction. In addition to these archived isolates, further isolates were obtained through sample testing associated with wildlife health surveillance projects. Various tissue samples from wild ungulates (moose, caribou and muskoxen) were selectively cultured for *E. rhusiopathiae*: 2 g of tissue were mechanically homogenized in 20 ml of brain heart infusion (BHI) broth with 5 % serum using a Stomacher 80 Biomaster (Seward, Port Saint Lucie, FL, USA), incubated overnight at 37 °C with 5 % CO_2_, followed by 48 h incubation in selective medium containing kanamycin (40 μg/ml), neomycin (50 μg/ml) and vancomycin (25 μg/ml) [60, 61] and sub-culture to agar plates of the same selective medium for 48-72 h at 37 °C with 5 % CO_2_. Colonies were further sub-cultured on CA plates as described for the other isolates.

### DNA extraction and PCR confirmation

DNA was extracted from clonal populations by suspending 1 loopful of colonies in 200 μl of phosphate buffered saline (PBS), and then using the DNeasy Blood and Tissue Kit (Qiagen, Mississauga, ON, Canada) following manufacturer’s instructions, with the exception that DNA was eluted in a smaller volume (50 μl) of the provided elution buffer to yield a higher DNA concentration. Extracted DNA was confirmed to be from *E. rhusiopathiae* by qPCR using previously described primers and a species-specific probe targeting the 3’ non-coding region of the rRNA gene cluster [27]. The 20 μl PCR reaction consisted of 10 μl of TaqMan Fast Advanced Master Mix (Applied Biosystems, Carlsbad, CA), 10 pmol of each primer, 1 pmol of probe, and 2 μl of template DNA. Reactions were performed using a CFX96 thermocycler (Bio-Rad, Mississauga, Ontario, Canada) with the following cycling conditions: 50 °C for 2 min, 95 °C for 20 s, then 40 cycles of 95 °C for 3 s and 57 °C for 30 s. *E. tonsillarum* isolates were also confirmed by probe-based qPCR using a different probe with this same set of primers and reaction conditions [27]. The identity of *E.* sp. strain 2 was confirmed *in silico* by identifying the specific primer sequences for the *E.* sp. strain 2 23S rRNA gene in the draft assembly [62].

### Library preparation and sequencing

Library preparation was performed using the Nextera XT v2 kit (Illumina, San Diego, CA) following manufacturer’s instructions, including a PhiX control spiked in at 1 %. Multiplex sequencing was performed on an Illumina MiSeq machine at the University of Calgary, resulting in 250 base pair paired-end reads.

### Read mapping and variant detection

The program ConDeTri was used to trim raw reads, extract those of high quality, and remove duplicates [63] (see Additional file 2: Figure S6 for a schematic diagram of the analysis pipeline, and Additional file 4: File S1 for specific commands and parameters used for each program). Unique trimmed reads from each isolate were mapped against the complete *E. rhusiopathiae* Fujisawa genome [Genbank:NC_015601] using BWA-MEM with default settings [64]. Sequencing and mapping parameters (number of reads, number of mapped reads, mean coverage, mean mapping quality) were extracted from .bam files using Qualimap [65] (Additional file 1: Table S1). All sequenced isolates had a minimum mean coverage of 9X, with an average mean coverage of 33X. Median mapping quality was consistently high, with a mean value of 57.

Variants were detected using the mpileup command in SAMtools [66]. A list of high quality SNP sites across all *E. rhusiopathiae* genomes was generated using custom python scripts (written by HT; available upon request) that filtered on base quality, mapping quality, read depth, and heterozygosity. A site was included in the variant list if it had consensus base quality ≥ 40, mapping quality ≥ 40, at least three reads mapping to that site on each strand, and the majority base present in >95 % of reads at that site for at least one isolate. Sites were excluded where more than one alternate allele was found since these positions were believed to be less phylogenetically reliable due to possible sequence saturation or mapping error. Alleles at each SNP site were called across all isolates if the consensus base quality was ≥ 30, mapping quality was ≥ 30, a minimum of 2 reads were present on each strand, and >95 % of reads supported the same allele in the isolate. Additional whole genome sequences of *E. rhusiopathiae* available on GenBank at the time of writing, strain SY1027 [Genbank:NC_021354] and the draft sequence of the strain ATCC19414 [Genbank:NZ_ACLK00000000] were included by simulating reads using the wgsim script in SAMtools and mapping these back to the reference genome in the same way as the other isolates for allele calling. Mobile and repetitive elements were identified in the annotated reference genome, as well as using the repeat-match command in MUMmer [67] and were removed from the variant list. A total of 32,148 high quality unique variant sites compared to the Fujisawa reference genome were identified across all 85 *E. rhusiopathiae* isolates (including the two GenBank sequences) once SNPs within repetitive regions and mobile elements were removed. A list of sites where alleles were correctly called in 100 % of isolates (*n* = 6078) was then generated. A total of 142 *E. rhusiopathiae* isolates were sequenced [Genbank BioProject: PRJNA288715], however in cases where multiple isolates were considered to be epidemiologically linked based on metadata and sequence similarity (i.e. <15 high quality SNP differences across the whole genome), only the isolate with the highest coverage was selected for inclusion in this analysis.

### *De novo* assembly

Unique, trimmed reads were assembled using SPAdes [68] applying a k-value of 55; this assembler was selected as the best compromise between contig number/L50 and fewer misassemblies based on metrics generated for candidate assemblers using QUAST [69]. SPAdes was run using the built-in BayesHammer for further read error correction [70] and Mismatch Corrector, a post processing tool that uses BWA. The PAGIT suite of programs was used to order and extend contigs and correct errors in the consensus sequence [71]. Output assembly metrics pre-PAGIT (number of contigs, N50) and post-PAGIT (number of scaffolds and GC %) were determined using QUAST (Additional file 1: Table S1). The average number of scaffolds per isolate after the PAGIT improvement pipeline was 13 (range: 4–89). The average GC% ranged from 35.9–36.5 %, with the mean across all isolates being 36.3 %.

### Annotation

RAST (Rapid Annotation using Subsystem Technology) was used to annotate and predict coding sequences in each isolate [72]. Putative prophage sequences were detected using PHAST [73]. To identify potential plasmid-associated sequences, contigs that were not aligned to the reference genome in PAGIT were searched against the NCBI database using Megablast. Additionally, a BLAST search of the *E. rhusiopathiae* plasmid sequence available on GenBank was performed against all assemblies. Hits to known plasmids or to genes encoding conjugal transfer proteins were considered suggestive of the presence of plasmids. To determine the Spa-type of each isolate, a custom BLAST database including representative sequences from the three recognized Spa types (A, B and C) [49] was searched against each *E. rhusiopathiae* isolate using Geneious version 7.1.8 [74]. The serotype of isolate VI11-2_lu was determined using methods previously described [56]. The genetic basis for differences in serotype has not been previously described in the literature, therefore it was not possible to determine the serotype of unknown isolates based on the genomic sequence data.

### Comparative genomics

Pan genome statistics were generated using LS-BSR [75] and visualized using PanGP [76]. To improve sensitivity in detecting core proteins that might be missed due to inferior assembly quality in isolates with a lower depth of coverage, isolates with coverage less than 15X (*n* = 8) were excluded from this analysis. The LS-BSR script compare_BSR.py was used to look for differences in gene content between groups of isolates. Specifically, pairwise comparisons in gene content (genes present in all members of one group that are absent in all members of a second group) were made among the different Clades (as determined by PhyloPhlAn and fineSTRUCTURE), between isolates originating from specific host species (swine, poultry, marine mammal and muskox isolates compared to all other isolates), as well as geographic origin (North American vs. European). Unfortunately, given the lack of metadata on clinical manifestation associated with our collection of isolates, it was not possible to assess genetic differences that might be associated with pathogenicity in this study. The core genetic content was visualized in GView using isolates with coverage ≥ 15X [77].

### Tests for recombination

The program Parsnp [78] was used to generate a core genome alignment (i.e. conserved orthologous regions present in all included genomes). Input for this alignment was the 83 PAGIT-improved *E. rhusiopathiae de novo* assemblies, as well as the three *E. rhusiopathiae* sequences available from GenBank. Putative prophage sequences detected with PHAST were masked using the BEDTools maskfasta command [79] prior to creating the alignment. This core nucleotide alignment was used as the input for both BratNextGen [80] and Gubbins [40]. Since Gubbins is best suited for detecting recombination in closely-related groups of isolates [40], separate analyses were run for each clade of isolates as later determined using PhyloPhlAn and fineSTRUCTURE. BratNextGen was run setting the hyperparameter α to 4 and using 20 iterations of the recombination estimation algorithm. The statistical significance was estimated using 100 permutations of the algorithm, setting significance at α = 0.05. Gubbins was run within the publicly available virtual machine using default settings. Recombination to mutation rates (r/m; number of SNPs in recombinogenic segments: number of SNPs inferred to be the result of spontaneous mutation) were calculated from Gubbins output by taking the sum of mutations inside and outside recombinant segments along all internal branches leading to each terminal node of the output tree, starting from the node of the inferred common ancestor of that clade.

### Examination of the population structure

Since we found that recombination has played an important role in generating the diversity observed within the species *E. rhusiopathiae*, three techniques that are robust to recombination were used to examine the population structure. First, PhyloPhlAn was used to estimate a phylogenetic tree based on a set of > 400 conserved protein sequences common to most bacteria [44]. The amino acid fasta files generated using RAST were used as the input for the program. The following isolates were included: 83 *de novo* and three *E. rhusiopathiae* strains available on GenBank, three *de novo* assembled *E.* spp*.* isolates (Additional file 1: Table S1), as well as the draft sequence of *E. tonsillarum* [NZ_AREO00000000]. As an outgroup, draft whole genome sequences of a selection of members from closely related genera [20] were retrieved from GenBank. These were of *Holdemania filiformis* DSM 12042 [NZ_ACCF00000000], *[Clostridium] spiroforme* DSM 1552 [NZ_ABIK00000000], *Erysipelatoclostridium ramosum* DSM 1402 [NZ_ABFX00000000], *Solobacterium moorei* F0204 [NZ_AECQ00000000], and *Bulleidia extructa* W1219 [NZ_ADFR00000000]. A circularized version of this tree was generated in the Interactive Tree of Life (iTOL) v3.0 to illustrate the relationship among the *E. rhusiopathiae* isolates and their host species and geographic origin [81].

ChromoPainter and fineSTRUCTURE were implemented as an alternative method for examining population structure without relying on phylogenetic inference [45]. This analysis assesses each genome (recipient) as the sum of segments that could have been received from any of the other genomes in the analysis (donors). These inferred shared segments are summarized in a ‘co-ancestry matrix’ through Principal Component Analysis which is then used as the input for Bayesian Markov chain Monte Carlo (MCMC) model-based clustering. We used the SNP alignment obtained from the reference-based mapping approach as the input for ChromoPainter, including all 86 *E. rhusiopathiae* isolates. Instructions in the example provided on the official program web page were followed [82]. A uniform recombination map was generated using a perl script available with this program, and was used in ChromoPainter’s E-M procedure for estimating the parameters of effective population size and global mutation rate (estimated to be 2379.89 and 1.17 E-3 respectively). These parameters were then used to run ChromoPainter, followed by ChromoCombine which calculates the variance expected in the data, needed for running fineSTRUCTURE. This was run using 1,000,000 MCMC iterations, half of which were burn-in, sampling every 250 iterations. A heat map representing the inferred relationships was generated using the fineSTRUCTURE graphical user interface.

Finally, a phylogenetic network was inferred using Neighbor-Net [46], implemented within SplitsTree using a core nucleotide alignment of all 86 *E. rhusiopathiae* genomes without masked phage sequences generated in Parsnp. To achieve greater resolution among the isolates of Clade 3 which was found to be the dominant clade, the SNP sites determined to be outside of recombinant segments by Gubbins (*n* = 7580) were used to generate a maximum likelihood tree of the isolates within this clade using PhyML [83], using a generalized time-reversible (GTR) model of nucleotide substitution with a gamma distribution, and performing 1000 bootstrap replicates. To test whether a temporal signal could be detected in Clade 3 isolates once recombinant segments had been removed, this maximum likelihood tree was examined in Path-O-Gen, which looks for correlation between year of isolation and root-to-tip divergence [84]. Clustering within Clade 3 by host species (swine/poultry/wild birds/wild ungulates/other), geographic origin (Europe, central North America, Northern Canadian provinces, Arctic, other/unknown) or submitting laboratory was examined in BaTS [85]. BaTS analysis was run on a set of trees generated in MrBayes [86], using the curated set of non-recombinant SNPs from Gubbins as the input alignment, performing 10 million iterations, sampling every 1000 samples, and taking the first half as burn-in. A Fisher’s exact test was used to assess the statistical significance of the lack of Clade 3 isolates found among marine mammals.

### Ethics approval

Sample collections from wild ungulate carcasses were approved by the University of Calgary Animal Care Committee (AC13-0072) which adheres to the guidelines of the Canadian Council on Animal Care.

### Availability of data

All raw sequence reads (Additional file 1: Table S1) are available on the NCBI Sequence Read Archive [BioProject PRJNA288715].

## Additional files

Additional file 1: Table S1. Excel spreadsheet of the *Erysipelothrix* spp. isolates used in this study, including metadata and assembly statistics. (XLSX 24 kb) Additional file 2: Supplementary figures. **Figure S1.** *Erysipelothrix rhusiopathiae* core genome. Core genes were plotted against the *E. rhusiopathiae* Fujisawa reference genome using GView, filtering out low-complexity sequences (e.g. repetitive regions). Bacteriophage sequences in the annotated reference genome and core genes associated with bacterial competence are highlighted. Publicly available *E. rhusiopathiae* isolates and *de novo* assembled isolates whose average depth of coverage was greater than 15X were included in this analysis. **Figure S2.** Recombinant fragments estimated in the *Erysipelothrix rhusiopathiae* core genome using BratNextGen. Presence of the same color block across multiple isolates within a column represents acquisition of the same recombinant segment; otherwise colors are arbitrary. **Figure S3.** Recombinant fragments in Clades 1, 2 and 3 respectively, estimated in Gubbins. Red blocks are recombinant fragments that have been inherited by multiple isolates, while blue fragments are unique to that isolate. **Figure S4.** Population subgroup assignment of *Erysipelothrix rhusiopathiae* isolates during chromosome painting using ChromoPainter/fineSTRUCTURE as shown in Fig. 4. **Figure S5.** Homoplasy associated with *Erysipelothrix rhusiopathiae* serotyping. This figure shows examples of homoplasy in serotypes 1a, 2 and 5, based on the same phylogenetic tree shown in Fig. 3. Labelled arrows show the locations of isolates of the different serotypes. Serotype was previously determined for all isolates shown here except VI11-2_Lu, which was serotyped in this study. **Figure S6.** Data analysis pipeline. (PDF 695 kb) Additional file 3: Table S2. Excel spreadsheet presenting cluster statistics for host species, geographic location, and submitting laboratory, as output from the program BaTS. (XLSX 11 kb) Additional file 4: File S1. Program versions and specific command line options. (TXT 4 kb)

## References

[1] Wilson DJ (2012). Insights from genomics into bacterial pathogen populations. PLoS Pathog.

[2] Pannekoek Y, Dickx V, Beeckman DSA, Jolley KA, Keijzers WC, Vretou E (2010). Multi locus sequence typing of *Chlamydia* reveals an association between *Chlamydia psittaci* genotypes and host species. PLoS One.

[3] Chin C-S, Sorenson J, Harris JB, Robins WP, Charles RC, Jean-Charles RR (2010). The origin of the Haitian cholera outbreak strain. New Engl J Med.

[4] Croucher NJ, Harris SR, Grad YH, Hanage WP (2013). Bacterial genomes in epidemiology--present and future. Philos Trans R Soc Lond B Biol Sci.

[5] Muellner P, Pleydell E, Pirie R, Baker M, Campbell D, Carter P, et al. Molecular-based surveillance of campylobacteriosis in New Zealand – from source attribution to genomic epidemiology. Euro Surveill. 2013;18. 23351655

[6] Martin DP, Beiko RG, Robinson DA, Falush D, Feil EJ (2010). Genetic recombination and bacterial population structure. Bacterial population genetics in infectious disease.

[7] Veraldi S, Girgenti V, Dassoni F, Gianotti R (2009). Erysipeloid: a review. Clin Exp Dermatol.

[8] Leighton FA, Williams ES, Barker I (2008). Erysipelothrix infection. Infectious diseases of wild mammals.

[9] Opriessnig T, Wood RL, Zimmerman JJ, Karriker LA, Ramirez A, Schwartz KJ, Stevenson GW (2012). Erysipelas. Diseases of swine.

[10] Bender JS, Irwin CK, Shen H-G, Schwartz KJ, Opriessnig T (2011). *Erysipelothrix* spp. genotypes, serotypes, and surface protective antigen types associated with abattoir condemnations. J Vet Diagn Invest.

[11] Kwok AH, Li Y, Jiang J, Jiang P, Leung FC (2014). Complete genome assembly and characterization of an outbreak strain of the causative agent of swine erysipelas – *Erysipelothrix rhusiopathiae* SY1027. BMC Microbiol.

[12] To H, Sato H, Tazumi A, Tsutsumi N, Nagai S, Iwata A (2012). Characterization of *Erysipelothrix rhusiopathiae* strains isolated from recent swine erysipelas outbreaks in Japan. J Vet Med Sci.

[13] Janßen T, Voss M, Kühl M, Semmler T, Philipp H-C, Ewers C (2015). A combinational approach of multilocus sequence typing and other molecular typing methods in unravelling the epidemiology of *Erysipelothrix rhusiopathiae* strains from poultry and mammals. Vet Res.

[14] Eriksson H, Nyman A-K, Fellström C, Wallgren P (2013). Erysipelas in laying hens is associated with housing system. Vet Rec.

[15] Boehm J, Lacave G, Patterson R, editors. Proceedings of the first international workshop on erysipelas in cetaceans. Chicago; 2000. 101 pp.

[16] Lacave G, Cox E, Hermans J, Devriese L, Goddeeris BM (2001). Induction of cross-protection in mice against dolphin *Erysipelothrix rhusiopathiae* isolates with a swine commercial vaccine. Vet Microbiol.

[17] Kutz S, Bollinger T, Branigan M, Checkley S, Davison T, Dumond M (2015). *Erysipelothrix rhusiopathiae* associated with recent widespread muskox mortalities in the Canadian Arctic. Can Vet J.

[18] Brooke CJ, Riley TV (1999). *Erysipelothrix rhusiopathiae*: bacteriology, epidemiology and clinical manifestations of an occupational pathogen. J Med Microbiol.

[19] Ogawa Y, Ooka T, Shi F, Ogura Y, Nakayama K, Hayashi T (2011). The genome of *Erysipelothrix rhusiopathiae*, the causative agent of swine erysipelas, reveals new insights into the evolution of firmicutes and the organism’s intracellular adaptations. J Bacteriol.

[20] Verbarg S, Göker M, Scheuner C, Schumann P, Stackebrandt E, Rosenberg E, DeLong EF, Lory S, Stackebrandt E, Thompson F (2014). The families Erysipelotrichaceae emend., Coprobacillaceae fam. nov., and Turicibacteraceae fam. nov. The prokaryotes.

[21] Takahashi T, Fujisawa T, Tamura Y, Suzuki S, Muramatsu M, Sawada T (1992). DNA relatedness among *Erysipelothrix rhusiopathiae* strains representing all twenty-three serovars and *Erysipelothrix tonsillarum*. Int J Syst Bacteriol.

[22] Verbarg S, Rheims H, Emus S, Frühling A, Kroppenstedt RM, Stackebrandt E (2004). *Erysipelothrix inopinata* sp. nov., isolated in the course of sterile filtration of vegetable peptone broth, and description of Erysipelotrichaceae fam. nov. Int J Syst Evol Microbiol.

[23] Bang B-H, Rhee M-S, Chang D-H, Park D-S, Kim B-C (2015). *Erysipelothrix larvae* sp. nov., isolated from the larval gut of the rhinoceros beetle, *Trypoxylus dichotomus* (Coleoptera: Scarabaeidae). Antonie Van Leeuwenhoek.

[24] Takahashi T, Sawada T, Muramatsu M, Tamura Y, Fujisawa T, Benno Y (1987). Serotype, antimicrobial susceptibility, and pathogenicity of *Erysipelothrix rhusiopathiae* isolates from tonsils of apparently healthy slaughter pigs. J Clin Microbiol.

[25] Takahashi T, Fujisawa T, Umeno A, Kozasa T, Yamamoto K, Sawada T (2008). A taxonomic study on *Erysipelothrix* by DNA-DNA hybridization experiments with numerous strains isolated from extensive origins. Microbiol Immunol.

[26] Kucsera G (1973). Proposal for standardization of the designations used for serotypes of *Erysipelothrix rhusiopathiae* (Migula) Buchanan. Int J Syst Bacteriol.

[27] Pal N, Bender JS, Opriessnig T (2010). Rapid detection and differentiation of *Erysipelothrix* spp. by a novel multiplex real-time PCR assay. J Appl Microbiol.

[28] King SJ, Leigh JA, Heath PJ, Luque I, Tarradas C, Dowson CG (2002). Development of a multilocus sequence typing scheme for the pig pathogen *Streptococcus suis*: identification of virulent clones and potential capsular serotype exchange. J Clin Microbiol.

[29] Ingebritson AL, Roth JA, Hauer PJ (2010). *Erysipelothrix rhusiopathiae*: association of Spa-type with serotype and role in protective immunity. Vaccine.

[30] Wang Q, Chang BJ, Riley TV (2010). *Erysipelothrix rhusiopathiae*. Vet Microbiol.

[31] Mell JC, Redfield RJ (2014). Natural competence and the evolution of DNA uptake specificity. J Bacteriol.

[32] Laurenceau R, Péhau-Arnaudet G, Baconnais S, Gault J, Malosse C, Dujeancourt A (2013). A type IV pilus mediates DNA binding during natural transformation in *Streptococcus pneumoniae*. PLoS Pathog.

[33] Edwards DJ, Holt KE (2013). Beginner’s guide to comparative bacterial genome analysis using next-generation sequence data. Microb Inform Exp.

[34] Noguchi N, Sasatsu M, Takahashi T, Ohmae K, Terakado N, Kono M (1993). Detection of plasmid DNA in *Erysipelothrix rhusiopathiae* isolated from pigs with chronic swine erysipelas. J Vet Med Sci.

[35] Eamens GJ, Forbes WA, Djordjevic SP (2006). Characterisation of *Erysipelothrix rhusiopathiae* isolates from pigs associated with vaccine breakdowns. Vet Microbiol.

[36] Vos M, Didelot X (2009). A comparison of homologous recombination rates in bacteria and archaea. ISME J.

[37] Croucher NJ, Harris SR, Fraser C, Quail MA, Burton J, van der Linden M (2011). Rapid pneumococcal evolution in response to clinical interventions. Science.

[38] Harris SR, Clarke IN, Seth-Smith HMB, Solomon AW, Cutcliffe LT, Marsh P (2012). Whole-genome analysis of diverse *Chlamydia trachomatis* strains identifies phylogenetic relationships masked by current clinical typing. Nat Genet.

[39] Grad YH, Kirkcaldy RD, Trees D, Dordel J, Harris SR, Goldstein E (2014). Genomic epidemiology of *Neisseria gonorrhoeae* with reduced susceptibility to cefixime in the USA: a retrospective observational study. Lancet Infect Dis.

[40] Croucher NJ, Page AJ, Connor TR, Delaney AJ, Keane JA, Bentley SD (2015). Rapid phylogenetic analysis of large samples of recombinant bacterial whole genome sequences using Gubbins. Nucl Acids Res.

[41] Darch SE, McNally A, Harrison F, Corander J, Barr HL, Paszkiewicz K (2015). Recombination is a key driver of genomic and phenotypic diversity in a *Pseudomonas aeruginosa* population during cystic fibrosis infection. Sci Rep.

[42] Langridge GC, Fookes M, Connor TR, Feltwell T, Feasey N, Parsons BN (2015). Patterns of genome evolution that have accompanied host adaptation in *Salmonella*. Proc Natl Acad Sci U S A.

[43] den Bakker HC, Didelot X, Fortes ED, Nightingale KK, Wiedmann M (2008). Lineage specific recombination rates and microevolution in *Listeria monocytogenes*. BMC Evol Biol.

[44] Segata N, Börnigen D, Morgan XC, Huttenhower C (2013). PhyloPhlAn is a new method for improved phylogenetic and taxonomic placement of microbes. Nat Commun.

[45] Lawson DJ, Hellenthal G, Myers S, Falush D (2012). Inference of population structure using dense haplotype data. PLoS Genet.

[46] Bryant D, Moulton V (2004). Neighbor-net: an agglomerative method for the construction of phylogenetic networks. Mol Biol Evol.

[47] Harada T, Ogawa Y, Eguchi M, Shi F, Sato M, Uchida K (2014). Phosphorylcholine and SpaA, a choline-binding protein, are involved in the adherence of *Erysipelothrix rhusiopathiae* to porcine endothelial cells, but this adherence is not mediated by the PAF receptor. Vet Microbiol.

[48] Borrathybay E, Gong F-J, Zhang L, Nazierbieke W (2015). Role of surface protective antigen A in the pathogenesis of *Erysipelothrix rhusiopathiae* strain C43065. J Microbiol Biotechnol.

[49] To H, Nagai S (2007). Genetic and antigenic diversity of the surface protective antigen proteins of *Erysipelothrix rhusiopathiae*. Clin Vaccine Immunol.

[50] Chooromoney KN, Hampson DJ, Eamens GJ, Turner MJ (1994). Analysis of *Erysipelothrix rhusiopathiae* and *Erysipelothrix tonsillarum* by multilocus enzyme electrophoresis. J Clin Microbiol.

[51] Eriksson H, Jansson DS, Johansson K-E, Båverud V, Chirico J, Aspán A (2009). Characterization of *Erysipelothrix rhusiopathiae* isolates from poultry, pigs, emus, the poultry red mite and other animals. Vet Microbiol.

[52] Pan X, Yang Y, Zhang J-R (2014). Molecular basis of host specificity in human pathogenic bacteria. Emerg Microbes Infect.

[53] Hotchkiss EJ, Hodgson JC, Lainson FA, Zadoks RN (2011). Multilocus sequence typing of a global collection of *Pasteurella multocida* isolates from cattle and other host species demonstrates niche association. BMC Microbiol.

[54] Delannoy CMJ, Crumlish M, Fontaine MC, Pollock J, Foster G, Dagleish MP (2013). Human *Streptococcus agalactiae* strains in aquatic mammals and fish. BMC Microbiol.

[55] Kirzinger MWB, Stavrinides J (2012). Host specificity determinants as a genetic continuum. Trends Microbiol.

[56] Opriessnig T, Shen HG, Bender JS, Boehm JR, Halbur PG (2013). *Erysipelothrix rhusiopathiae* isolates recovered from fish, a harbour seal (*Phoca vitulina*) and the marine environment are capable of inducing characteristic cutaneous lesions in pigs. J Comp Pathol.

[57] Wellmann GG (1955). Summaries of experiments in swine erysipelas in Germany. JAVMA.

[58] Muellner P, Stärk KDC, Dufour S, Zadoks RN. “Next-generation” surveillance: an epidemiologists’ perspective on the use of molecular information in food safety and animal health decision-making. Zoonoses Public Health. 2015;e-pub ahead of print. 10.1111/zph.12230 26537766

[59] Brazelton WJ, Morrill PL, Szponar N, Schrenk MO (2013). Bacterial communities associated with subsurface geochemical processes in continental serpentinite springs. Appl Environ Microbiol.

[60] Wood RL (1965). A selective liquid medium utilizing antibiotics for isolation of *Erysipelothrix insidiosa*. Am J Vet Res.

[61] Bender JS, Kinyon JM, Kariyawasam S, Halbur PG, Opriessnig T (2009). Comparison of conventional direct and enrichment culture methods for *Erysipelothrix* spp. from experimentally and naturally infected swine. J Vet Diagn Invest.

[62] Takeshi K, Makino S, Ikeda T, Takada N, Nakashiro A, Nakanishi K (1999). Direct and rapid detection by PCR of *Erysipelothrix* sp. DNAs prepared from bacterial strains and animal tissues. J Clin Microbiol.

[63] Smeds L, Künstner A (2011). ConDeTri - a content dependent read trimmer for Illumina data. PLoS One.

[64] Li H. Aligning sequence reads, clone sequences and assembly contigs with BWA-MEM. Oxford University Press. 2013;arXiv:1303.3997.

[65] García-Alcalde F, Okonechnikov K, Carbonell J, Cruz LM, Götz S, Tarazona S (2012). Qualimap: evaluating next-generation sequencing alignment data. Bioinformatics.

[66] Li H, Handsaker B, Wysoker A, Fennell T, Ruan J, Homer N (2009). The sequence alignment/map format and SAMtools. Bioinformatics.

[67] Kurtz S, Phillippy A, Delcher AL, Smoot M, Shumway M, Antonescu C (2004). Versatile and open software for comparing large genomes. Genome Biol.

[68] Bankevich A, Nurk S, Antipov D, Gurevich AA, Dvorkin M, Kulikov AS (2012). SPAdes: a new genome assembly algorithm and its applications to single-cell sequencing. J Comput Biol.

[69] Gurevich A, Saveliev V, Vyahhi N, Tesler G (2013). QUAST: quality assessment tool for genome assemblies. Bioinformatics.

[70] Nikolenko SI, Korobeynikov AI, Alekseyev MA (2013). BayesHammer: Bayesian clustering for error correction in single-cell sequencing. BMC Genomics.

[71] Swain MT, Tsai IJ, Assefa SA, Newbold C, Berriman M, Otto TD (2012). A post-assembly genome-improvement toolkit (PAGIT) to obtain annotated genomes from contigs. Nat Protoc.

[72] Overbeek R, Olson R, Pusch GD, Olsen GJ, Davis JJ, Disz T (2014). The SEED and the rapid annotation of microbial genomes using subsystems technology (RAST). Nucleic Acids Res.

[73] Zhou Y, Liang Y, Lynch KH, Dennis JJ, Wishart DS (2011). PHAST: a fast phage search tool. Nucl Acids Res.

[74] Kearse M, Moir R, Wilson A, Stones-Havas S, Cheung M, Sturrock S (2012). Geneious basic: an integrated and extendable desktop software platform for the organization and analysis of sequence data. Bioinformatics.

[75] Sahl JW, Caporaso JG, Rasko DA, Keim P (2014). The large-scale blast score ratio (LS-BSR) pipeline: a method to rapidly compare genetic content between bacterial genomes. PeerJ.

[76] Zhao Y, Jia X, Yang J, Ling Y, Zhang Z, Yu J (2014). PanGP: A tool for quickly analyzing bacterial pan-genome profile. Bioinformatics.

[77] Petkau A, Stuart-Edwards M, Stothard P, Domselaar GV (2010). Interactive microbial genome visualization with GView. Bioinformatics.

[78] Treangen TJ, Ondov BD, Koren S, Phillippy AM (2014). Rapid core-genome alignment and visualization for thousands of microbial genomes. Genome Biol.

[79] Quinlan AR (2014). BEDTools: the swiss-army tool for genome feature analysis. Curr Protoc Bioinformatics.

[80] Marttinen P, Hanage WP, Croucher NJ, Connor TR, Harris SR, Bentley SD (2012). Detection of recombination events in bacterial genomes from large population samples. Nucl Acids Res.

[81] Letunic I, Bork P (2011). Interactive tree of life v2: online annotation and display of phylogenetic trees made easy. Nucleic Acids Res.

[82] Lawson D, Hellenthal G, Falush D, Myers S. PaintMyChromosomes.com: fineSTRUCTURE v2 & GLOBETROTTER. http://www.paintmychromosomes.com (2012). Accessed 14 Apr 2015.

[83] Guindon S, Gascuel O (2003). A simple, fast, and accurate algorithm to estimate large phylogenies by maximum likelihood. Syst Biol.

[84] Rambaut A. Molecular evolution, phylogenetics and epidemiology: Path-O-Gen. http://tree.bio.ed.ac.uk/software/pathogen (2007). Accessed 28 Apr 2015.

[85] Parker J, Rambaut A, Pybus OG (2008). Correlating viral phenotypes with phylogeny: accounting for phylogenetic uncertainty. Infect Genet Evol.

[86] Ronquist F, Teslenko M, van der Mark P, Ayres DL, Darling A, Höhna S (2012). MrBayes 3.2: efficient Bayesian phylogenetic inference and model choice across a large model space. Syst Biol.

