# Supplementary figures and images for: Genomic analysis of the multi-host pathogen Erysipelothrix rhusiopathiae reveals extensive recombination as well as the existence of three generalist clades with wide geographic distribution
Source: BMC Genomics. 2016 Jun 14;17:461. doi: 10.1186/s12864-016-2643-0 (PMC4906694; doi:10.1186/s12864-016-2643-0)

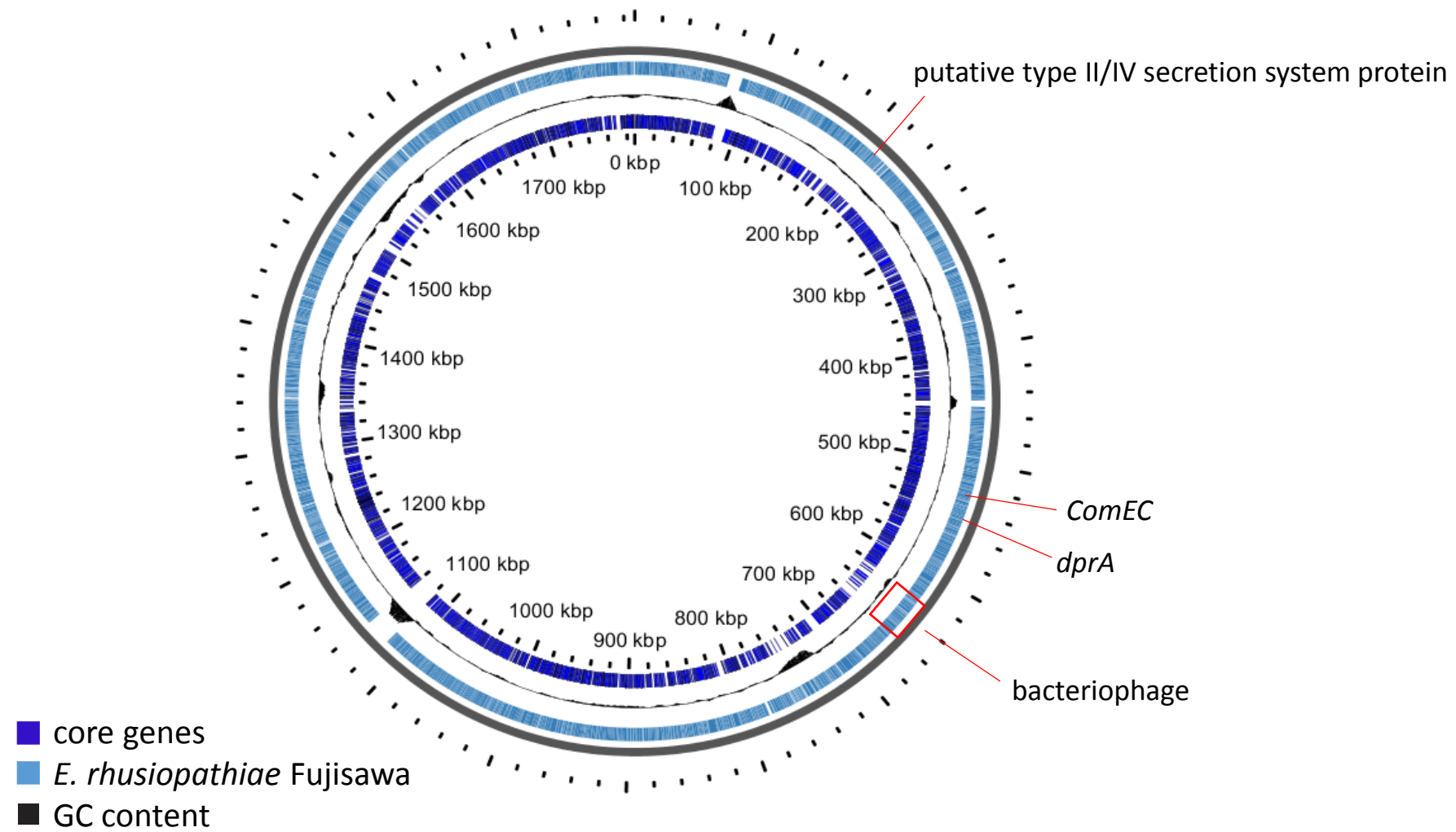

Figure S1

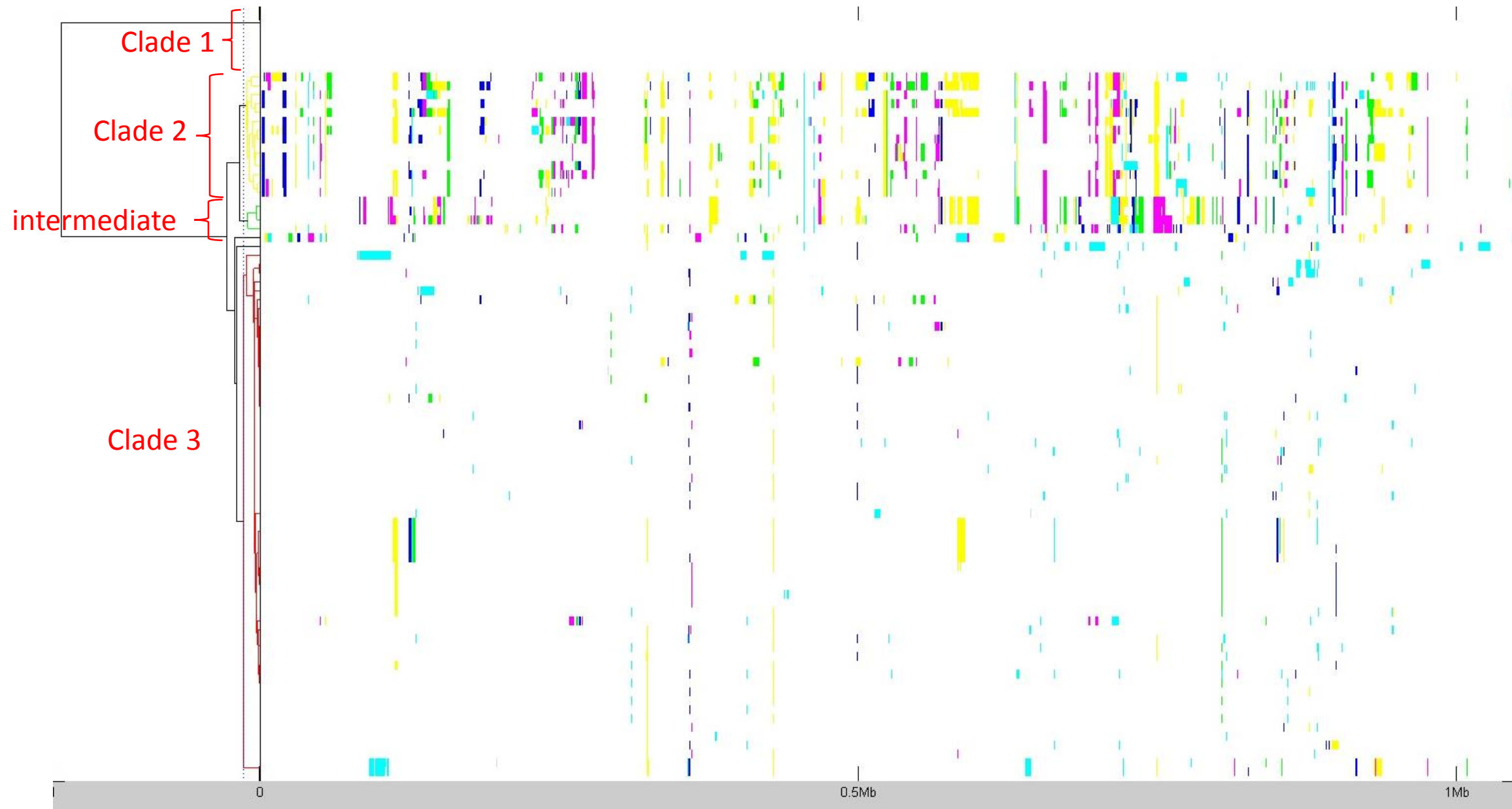

Figure S2

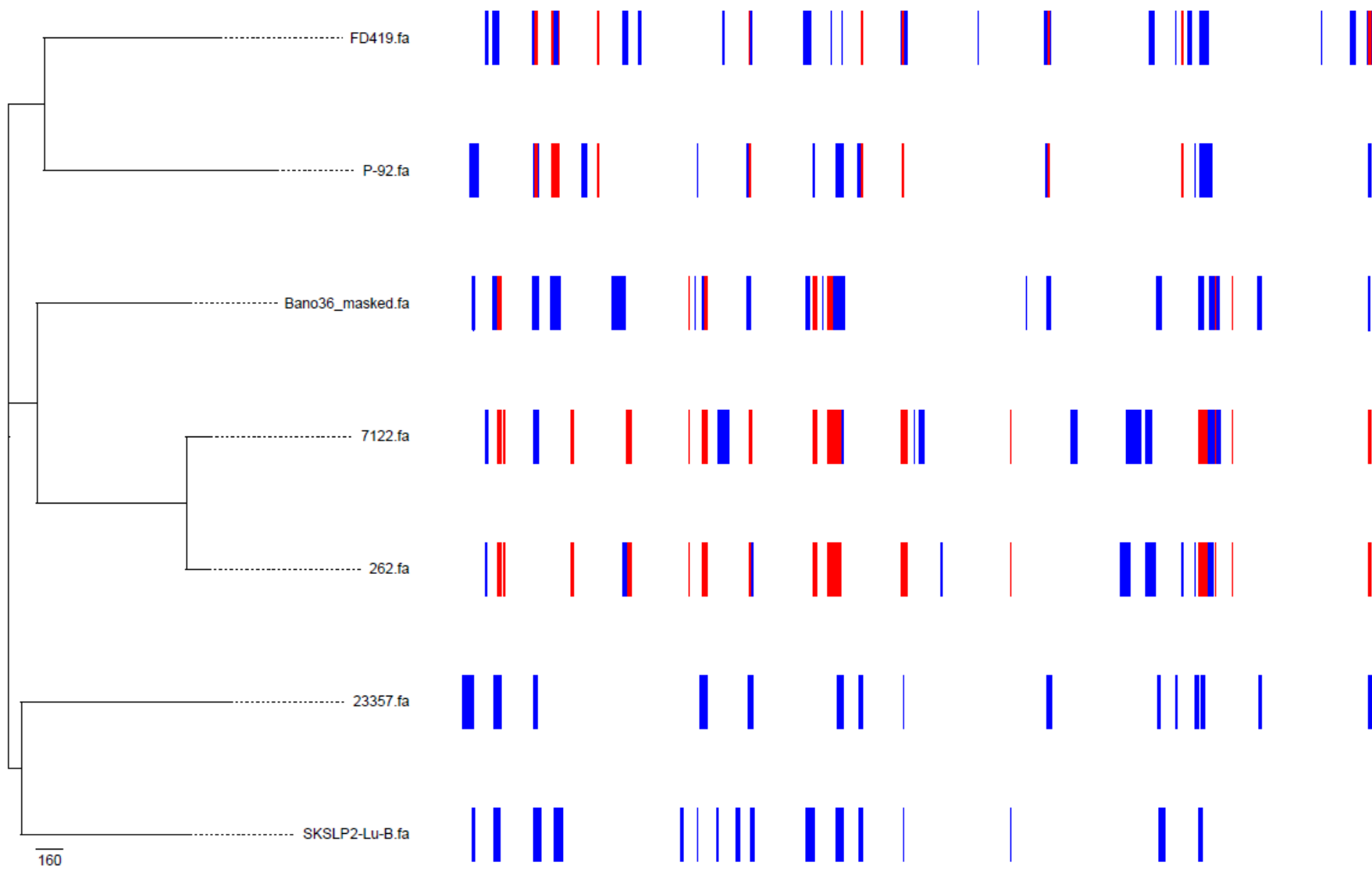

Figure S3- Clade 1

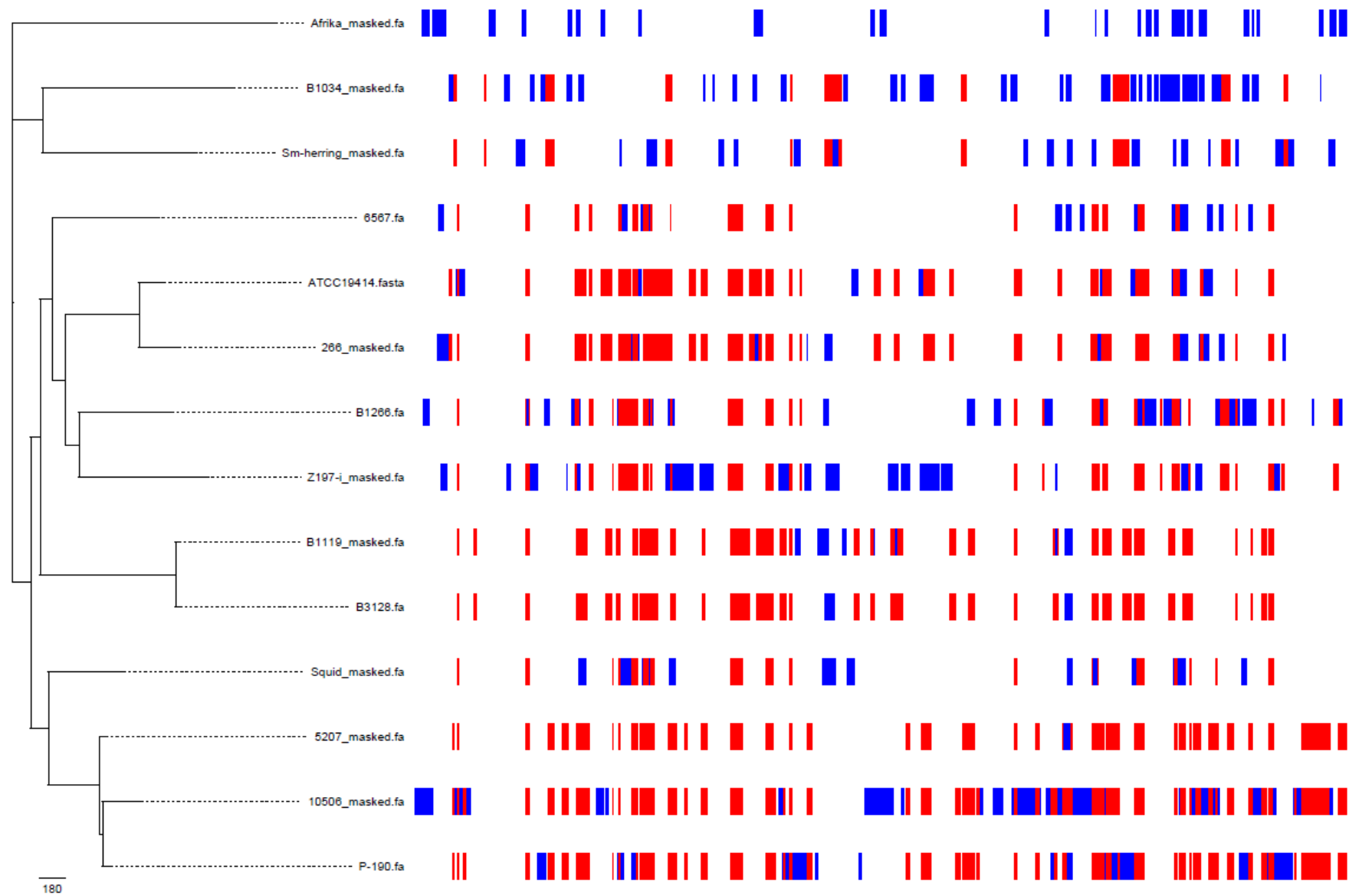

Figure S3- Clade 2

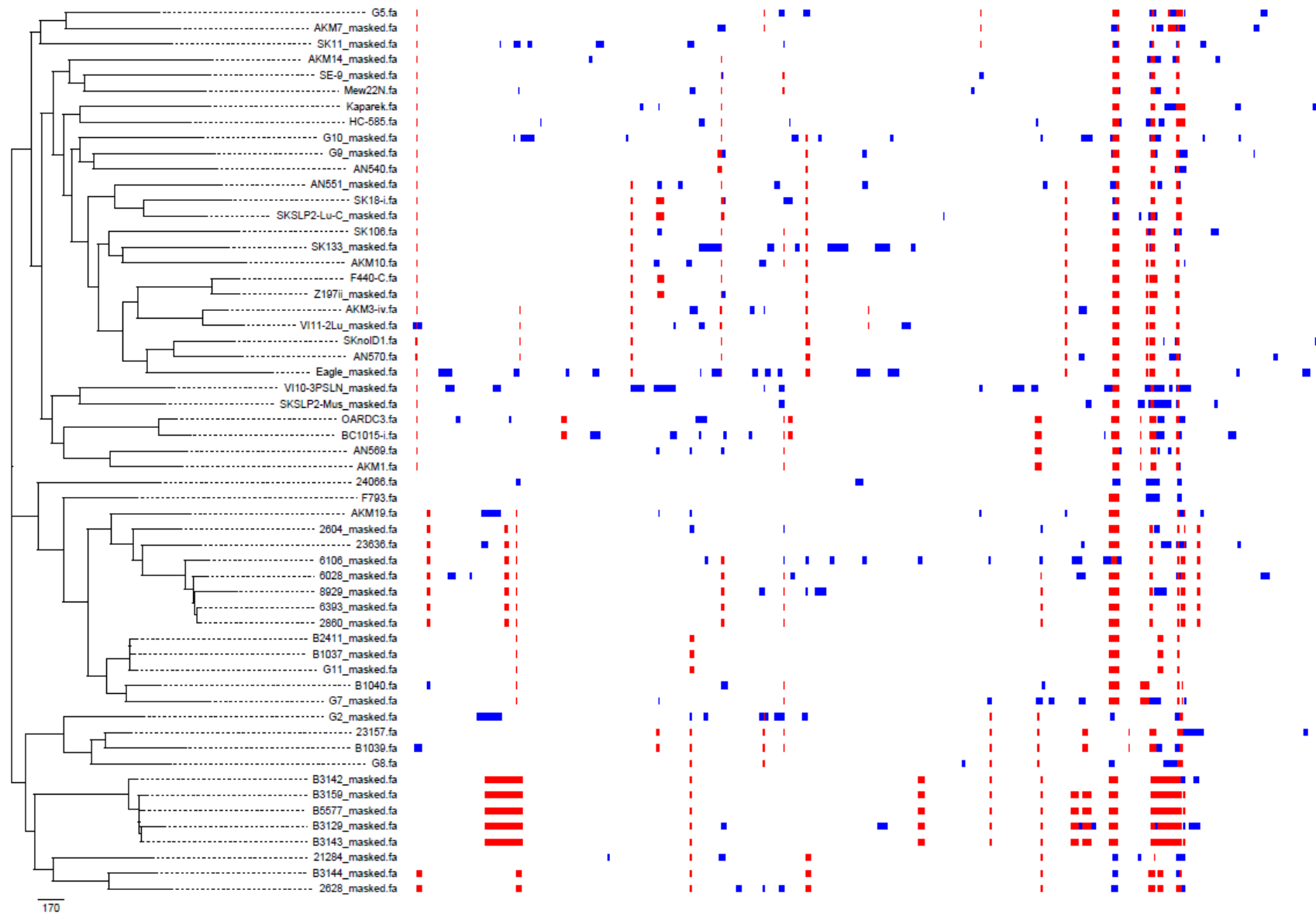

Figure S3- Clade 3

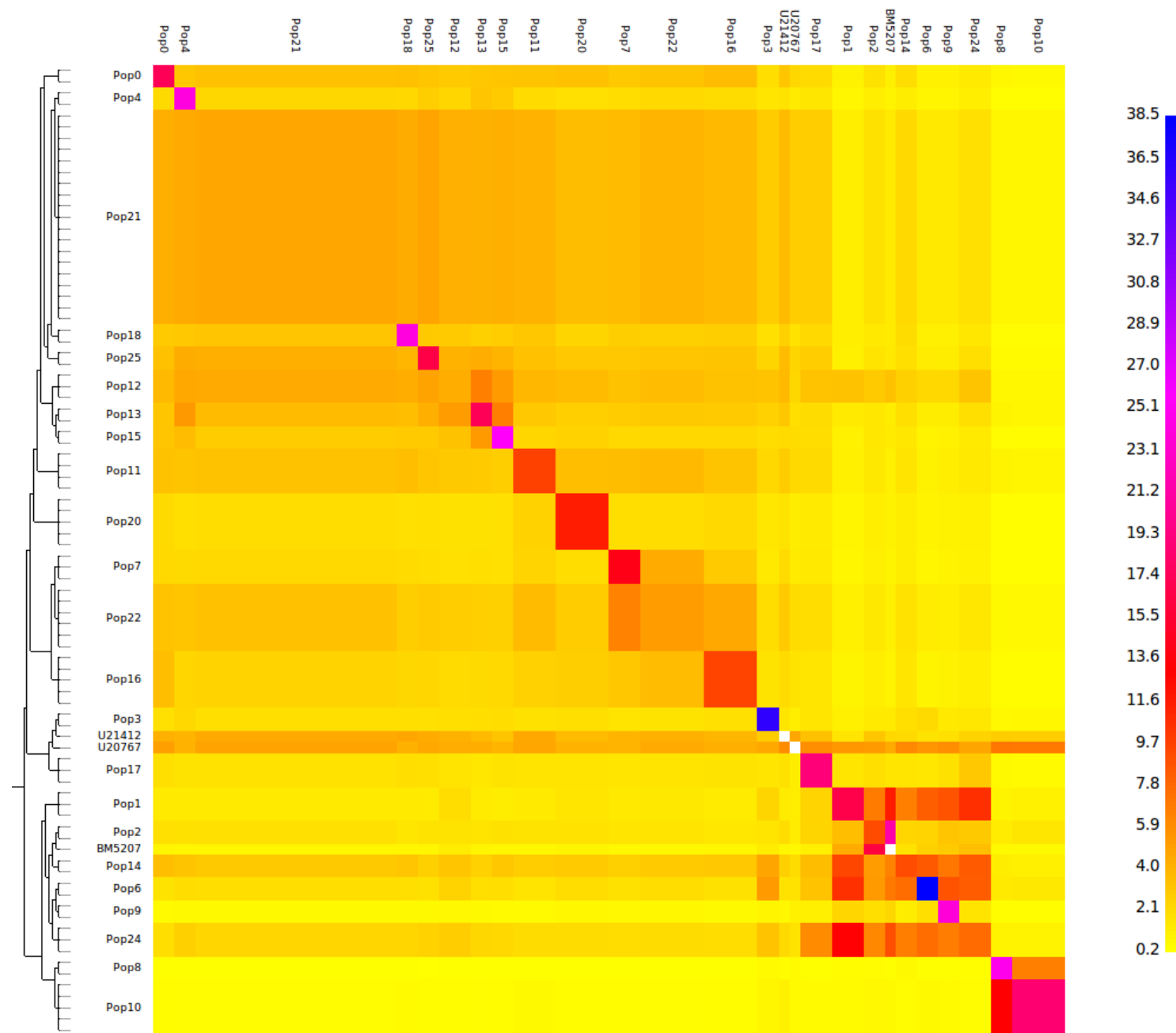

Figure S4

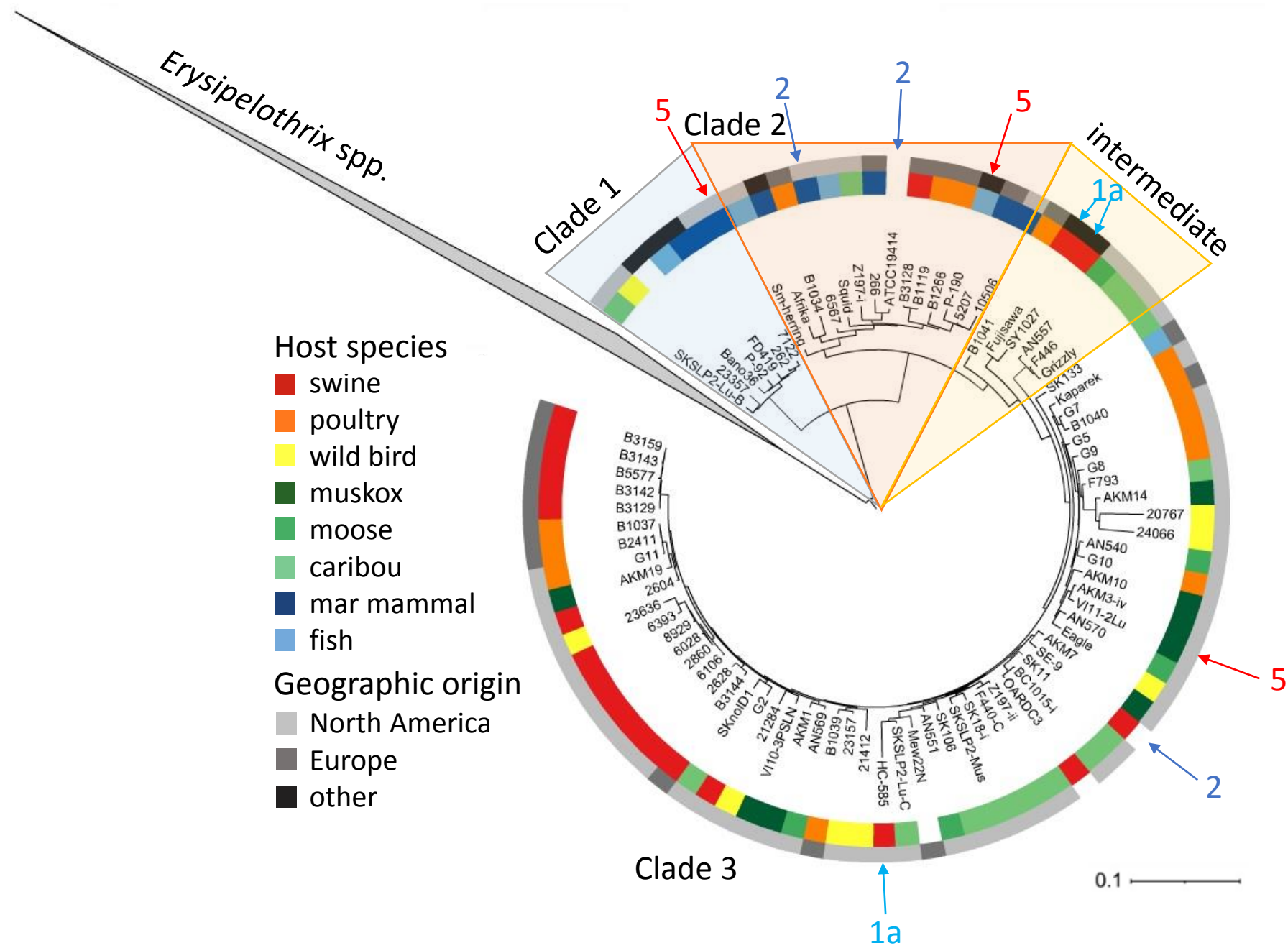

Figure S5

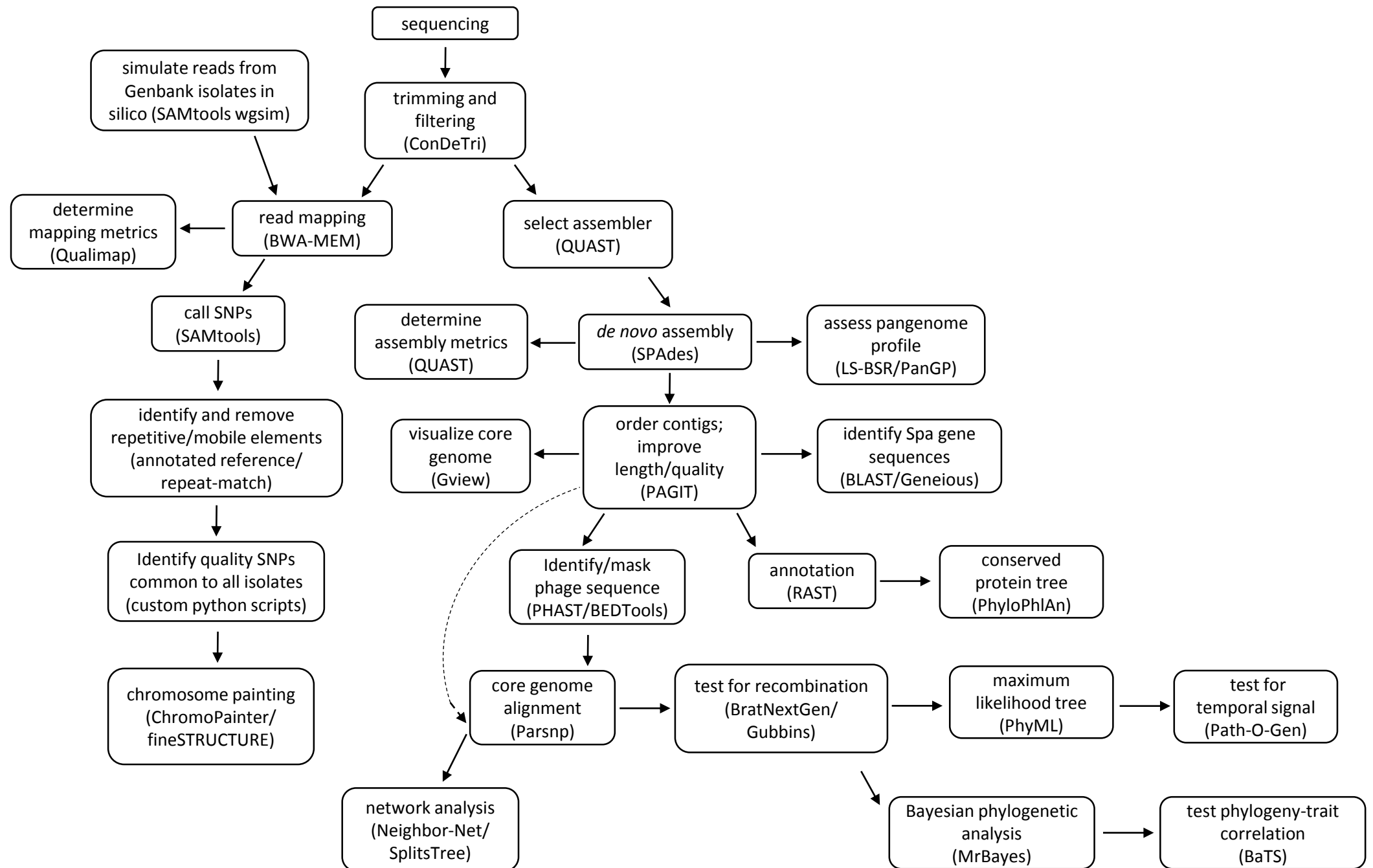

Figure S6

Supplement: Additional file 2: — Supplementary figures. Figure S1. Erysipelothrix rhusiopathiae core genome. Core genes were plotted against the E. rhusiopathiae Fujisawa reference genome using GView, filtering out low-complexity sequences (e.g. repetitive regions). Bacteriophage sequences in the annotated reference genome and core genes associated with bacterial competence are highlighted. Publicly available E. rhusiopathiae isolates and de novo assembled isolates whose average depth of coverage was greater than 15X were included in this analysis. Figure S2. Recombinant fragments estimated in the Erysipelothrix rhusiopathiae core genome using BratNextGen. Presence of the same color block across multiple isolates within a column represents acquisition of the same recombinant segment; otherwise colors are arbitrary. Figure S3. Recombinant fragments in Clades 1, 2 and 3 respectively, estimated in Gubbins. Red blocks are recombinant fragments that have been inherited by multiple isolates, while blue fragments are unique to that isolate. Figure S4. Population subgroup assignment of Erysipelothrix rhusiopathiae isolates during chromosome painting using ChromoPainter/fineSTRUCTURE as shown in Fig. 4. Figure S5. Homoplasy associated with Erysipelothrix rhusiopathiae serotyping. This figure shows examples of homoplasy in serotypes 1a, 2 and 5, based on the same phylogenetic tree shown in Fig. 3. Labelled arrows show the locations of isolates of the different serotypes. Serotype was previously determined for all isolates shown here except VI11-2_Lu, which was serotyped in this study. Figure S6. Data analysis pipeline. (PDF 695 kb) [file 12864_2016_2643_MOESM2_ESM.pdf]
